# Dimethyl‐, Diethyl‐, and Propylene Carbonates: An Emerging Class of Green Solvents for Organic Synthesis

**DOI:** 10.1002/tcr.202500350

**Published:** 2026-04-20

**Authors:** Gabriela T. Quadros, Livia C. L. Valente, Thiago Barcellos, Daniela Hartwig, Laura Abenante, Eder J. Lenardão

**Affiliations:** ^1^ Laboratório de Síntese Orgânica Limpa (LASOL‐CCQFA) Universidade Federal de Pelotas (UFPel) Pelotas RS Brazil; ^2^ Laboratório de Biotecnologia de Produtos Naturais e Sintéticos Universidade de Caxias do Sul Caxias do Sul RS Brazil

**Keywords:** dimethyl carbonate, ethyl carbonate, green solvents, organic carbonates, organic synthesis

## Abstract

The growing demand for green solvents to replace petroleum‐derived counterparts in organic synthesis has garnered significant attention, driven by the need for more sustainable production processes. In this context, organic carbonates (OCs) have emerged as prominent alternatives to toxic solvents that pose risks to human health and the environment. This review presents recent advances in the application of major dialkyl carbonates, namely dimethyl carbonate (DMC), diethyl carbonate (DEC), and propylene carbonate (PC), as solvents in organic synthesis. By discussing more than seventy papers from the past 6 years, we demonstrate the rising importance of this solvent class, which has transitioned from exotic adjuvants to essential components of modern synthetic methodologies. Through a compilation and analysis of the recent literature, this review aims to serve as a comprehensive resource for synthetic chemists, underscoring the potential of OCs to pave the way for safer and more sustainable synthesis.

## Graphical abstract

This review highlights recent advances in the use of organic carbonates, dimethyl carbonate (DMC), diethyl carbonate (DEC), and propylene carbonate (PC), as solvents in organic synthesis. Based on over seventy studies from the past 6 years, it shows their application in different organic reaction types, emphasizing their role in safer and more sustainable synthesis.

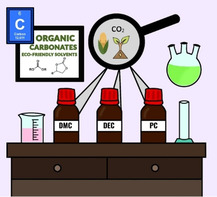

## 1 Introduction

Noteworthy, solvents are indispensable in most chemical reactions due to their role in dissolving solid substrates, facilitating mass and heat transfer, and influencing selectivity, yield, and the stabilization of intermediates [1, 2]. Furthermore, they are fundamental in substance extraction, chemical formulations, and purification processes, which require large volumes of them [1, 2]. The amount of solvents used in these processes can be estimated by the size of the market projected for the coming years. The global solvents market was valued at US$ 38.60 billion in 2024 and is expected to reach a valuation of US$ 61.95 billion by 2032 [3].

The increase in solvent consumption in various segments of the chemical industry, including the pharmaceutical and paint and coatings industries, comes at a price: a significant increase in chemical waste [1]. For example, the pharmaceutical industry is well known for using classic organic solvents throughout the entire drug manufacturing process, from synthesis to molecule purification. The raw materials needed to prepare active pharmaceutical ingredients (APIs) may contain up to 85% solvent by mass. Most classic organic solvents are derived from petroleum refining, are neither renewable nor biodegradable, and exhibit high toxicity, making them harmful to human health and theenvironment [1, 4, 5, 6].

Given the need to use solvents, but aware of the risks to human health and the environment, it is extremely necessary to make chemical processes more sustainable and environmentally benign. To this end, the concept of Green Chemistry emerges, aiming to encourage the use of renewable raw materials, to eliminate waste, and to avoid the use of toxic and/or hazardous reagents and solvents in the manufacture and application of chemical products. Therefore, the Twelve Principles of Green Chemistry serve as a guide for reducing waste generation, improving energy efficiency, and ensuring the safety of workers and handlers [4, 6, 7, 8, 9].

Four of the twelve principles are particularly relevant in the context of solvents: principle #1 is about prevention, which involves avoiding the use of harmful solvents and making the process residue‐free or clean; principle #5 focuses on using safer solvents and auxiliaries, which should ideally be avoided, but if they are necessary, they should be harmless; principle #7 emphasizes the use of renewable, biobased raw materials, recommending the use of renewable feedstocks instead of fossil sources. Finally, principle #12, which focuses on designing chemicals and processes to minimize the potential for accidents and releases [7, 8, 9].

In the face of the urgent need to preserve land, water, and soil, and with the goal of making chemical processes more eco‐friendly, the chemical and pharmaceutical industries have generally invested in developing sustainable alternatives that reduce the environmental impact of their activities. Considering this, green chemistry has introduced metrics to help industry and academia minimize their environmental impacts. One such metric is the E‐Factor (Environmental Factor), introduced by Roger Sheldon in 1992 [4]. This metric quantifies the amount of waste generated per kilogram of product and is based on preventing waste generation. There are also solvent guides, developed mainly by the pharmaceutical industry, that classify solvents according to their sustainability, safety, and health hazard characteristics, influencing the choice of greener solvents [6].

Consequently, considering the four principles of Green Chemistry, which are intrinsically linked to the use of organic solvents, alternative solvents are emerging to replace traditional ones, benefiting both industry and academia. These alternative solvents, also called green solvents, must have characteristics that make them more sustainable than conventional solvents. These characteristics include recyclability, low toxicity, low flammability, and biodegradability. Their use should also not result in the formation of undesirable byproducts [3, 5, 7, 8, 9].

In relation to this topic, we recently published a review article on the use of biobased solvents in organic synthesis, including carbohydrate‐ and terpene‐based solvents, as well as natural deep eutectic solvents [5]. In this context, organic carbonates (OCs) are another class of emerging green solvents that have attracted the attention of academia and industry, as they are used as solvents and reagents, acting as methylation and carboxymethylation agents, in the case of dimethyl carbonate (DMC).They are also already available on the Global market in wide quantities and at reasonable prices [10, 11]. Thus, this review article presents and discusses the most recent advances in the use of OCs as green solvents in organic reactions.

### 1.1 Chemical and Physicochemical Properties of OCs

**FIGURE 1:**
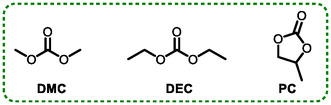
Chemical structures of DMC, DEC, and PC.

OCs, also known as carbonic acid esters, are a class of compounds with a carbonyl group flanked by two alkoxy/aryloxy groups. Their structures are represented by the formula R^1^OCOOR^2^, where R^1^ and R^2^ may be identical, or different alkyl or aryl groups, or they may form a cyclic structure. They are classified into linear carbonates (acyclic) and cyclic carbonates [11]. The OCs covered in this review article are linear dimethyl carbonate (DMC) and diethyl carbonate (DEC), and cyclic propylene carbonate (PC) (Figure 1).

The OCs exhibit different properties depending on their chemical structure. For example, cyclic OCs have a higher dielectric constant and boiling point compared to linear ones. Therefore, PC, in particular, is suitable for anhydrous electrochemical applications (Table 1) [10, 12]. However, PC has a high viscosity, which is disadvantageous for this type of application; therefore, a viable alternative is mixing it with another less viscous solvent to facilitate its use. In general, the use of solvent mixtures is a common and strategic practice, serving to optimize physicochemical properties such as polarity, viscosity, and solvation capacity [2].

**TABLE 1:** Physical‐chemical properties of OCs [10, 12].

	DMC	DEC	PC
Molecular weight, g mol^−1^	90.08	118.13	102.09
Melting point, °C	4.6	−43	−49
Boiling point, °C	90	126	243
Density, g cm^−3^	1.069	0.975	1.201
Water solubility, g L^−1^	139	insoluble	200
Dielectric constant, *ε*	3.20	2.82	66.6
Viscosity, mPa s^−1^	0.585 (25°C)	0.837 (20°C)	2.471–2.540 (25°C)
CAS number	616‐38‐6	105‐58‐8	108‐32‐7

The processing and purification of OCs, as well as the isolation of products in reactions that use them as a reaction medium, involve standardized laboratory and industrial methods. For OCs with low boiling points, the process is facilitated; however, for those with high boiling points, such as PC, it requires more specific techniques, such as distillation, which can also be applied at an industrial level [2, 13].

Most OCs are described as “readily biodegradable,” exhibiting high degradation rates in the environment over a short period of time, which is very advantageous for classifying them as environmentally safe and green solvents [12]. It is possible to compare the biodegradation rates of OCs with conventional solvents, such as tetrahydrofuran (THF), which has a biodegradation rate of 39% in 28 days, resulting in a classification of “not readily biodegradable,” while dichloromethane (CH_2_Cl_2_) has a biodegradation rate of 68% in 28 days, being classified as “readily biodegradable.” Nevertheless, even though it has the same classification as OCs, CH_2_Cl_2_ also falls into the class of halogenated solvents, meaning that other criteria need to be taken into consideration (Table 2) [10, 11, 12].

**TABLE 2:** Toxicologic properties of DMC, DEC, PC, DCM, and THF [10, 11, 12].

	DMC	DEC	PC	DCM	THF
Acute toxicity oral (rat)	LD_50_ > 5.000 mg kg^−1^	LD_50_ > 4.876 mg kg^−1^	LD_50_ > 5.000 mg kg^−1^	LD_50_ > 2.000 mg kg^−1^	LD_50_ 1.650 mg kg^−1^
Acute toxicity inhalation (rat or mouse)	LD_50_ > 5.36 mg L^−1^ (rat, 4 h)	No data available	No data available	LD_50_ 86 mg L^−1^ (mouse, 4 h)	LD_50_ > 14,7 mg L^−1^ (rat, 6 h)
Acute toxicity dermal (rabbit)	LD_50_ > 2.000 mg kg^−1^	No data available	LD_50_ > 2.000 mg kg^−1^	LD_50_ > 2.000 mg kg^−1^	LD_50_ > 2.000 mg kg^−1^
Biodegradability (aerobic)	Readily biodegradable 28 days ‐ 86%	Readily biodegradable 27 days ‐ 75%	Readily biodegradable 29 days – 87.7%	Readily biodegradable 28 days ‐ 68%	No treadily biodegradable 28 days ‐ 39%

### 1.2 Green Features

Currently, OCs may have an advantage over other green solvents due to their well‐established industrial‐scale production processes [14], thereby enabling them to meet the demand necessary for replacing conventional solvents at a relatively lower cost, in addition to their low toxicity and high biodegradability. Dimethyl carbonate (DMC) is the leading carbonate by production, with an estimated market size of US$ 937.74 million in 2025 and projected to reach US$ 1.5 billion by 2032 [15]. The increase in dimethyl carbonate production is driven by its use in the production of polycarbonates, as well as by the growing demand for electrolytic solvents in lithium battery manufacturing due to its high dielectric constant [3, 16]. Diethyl carbonate (DEC) and propylene carbonate (PC) also play a leading role as electrolytic solvents [2].

The main industrial production routes for OCs use carbon dioxide (CO_2_) as an initial input, which is considered a sustainable carbon source. For example, one of the most widely used processes in the synthesis of DMC involves the insertion of CO_2_ into epoxides and a subsequent transesterification reaction with methanol [10, 17, 18, 19]. Therefore, OCs add value to CO_2_ as a chemical input and can be considered carbon‐neutral or negative emission chemicals [17]. Due to the use of CO_2_ as a raw material and its high biodegradability rate, OCs are considered green solvents and have a minimal environmental impact after disposal.

In the field of organic reactions, OCs, especially DMC, DEC, and PC, have demonstrated excellent versatility as solvents, replacing traditional volatile and polar aprotic organic solvents, such as tetrahydrofuran (THF), acetonitrile (MeCN), dichloromethane (DCM), and aprotic ones, like dimethyl sulfoxide (DMSO), and dimethylformamide (DMF) [2, 10, 11].

While this article was being prepared, an excellent review covering the role of OCs as solvents in polymerization and depolymerization reactions, CO_2_ capture, membrane, films and fibers, materials and nanoparticles preparation, as well as uses in organic synthesis, extraction, analytical chemistry and other applications, has been published [10]. Regarding organic transformations, however, it was limited to part of the literature from 2007 to 2022, focusing on transition metal‐catalyzed reactions.

In this scenario, this review article presents and discusses the latest advances in the use of OCs as green solvents in organic synthesis. The focus is on the use of DMC, DEC, and PC, which are commercially available at relatively lowprices. Therefore, they are viable solvents that may, in the short term, become commonplace on laboratory shelves, like so many other conventional solvents.

This review surveyed the literature from 2019 to 2025, covering 65 synthetic methods, including coupling reactions, oxidations, reductions, and photocatalytic processes. A literature search was conducted on the Web of Science and Science Direct databases using relevant keywords, such as “OCs”, “solvents”, and “organic synthesis”. The resulting publications were then filtered to exclude those in which carbonates were used only in auxiliary procedures, like extraction or purification. The reviewed literature is presented in four main categories: (1) OCs in photo‐driven reactions; (2) OCs in metal‐catalyzed reactions; (3) OCs in metal‐free catalysis; and (4) OCs in other reactions, such as esterification, phosphorylation, multicomponent, cascade reactions, and so on.

## 2 OCs in Photo‐Driven Reactions

**SCHEME 1:**
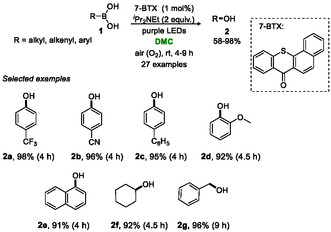
Visible light‐promoted oxidative hydroxylation of boronic acids **1** [20].

Ding et al. [20] published an oxidative hydroxylation of boronic acids **1** mediated by visible light (purple LEDs) using dimethyl carbonate as green solvent instead of the common solvent for this reaction, which is dichloromethane. The best reaction condition was using 7*H*‐benzo[*c*]thioxanthen‐7‐one (7‐BTX, 1 mol%) as a catalyst in the presence of 2 equiv. of *i*‐Pr_2_NEt, under an air atmosphere, for 4–9 h (Scheme 1). The targeted compounds **2** were obtained in 58–98% yield, and the protocol worked smoothly with a wide range of boronic acids **1**, including alkyl‐, alkenyl‐, and arylboronic ones. Unfortunately, heteroaromatic derivatives, such as furyl, thienyl, and pyridinyl boronic acids, did not react. Additionally, a gram‐scale reaction was carried out with phenylboronic acid as the starting material, affording the expected phenol in 87% yield after a simple recrystallization.

**SCHEME 2:**
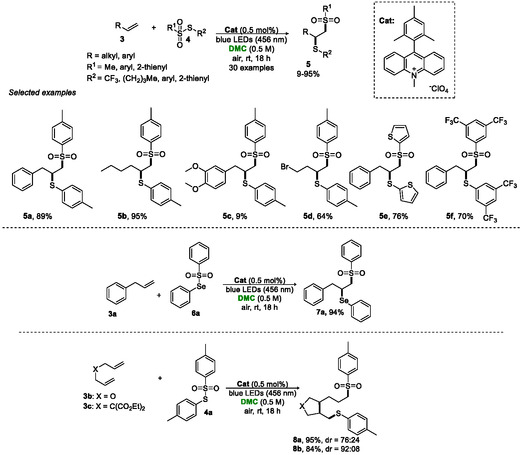
Blue LEDs irradiation in the vicinal thiosulfonylation reaction [21].

In 2020, Gadde et al. [21] described a green protocol metal‐ and oxidant‐free, under blue LEDs light irradiation to allow the vicinal thiosulfonylationreaction between nonactivated alkenes **3** (0.5 mmol) and thiosulfonates **4** (2 equiv.) (Scheme 2). The best reaction condition was found using 9‐mesityl‐10‐methylacridinium perchlorate as photocatalyst (0.5 mol%), in dimethyl carbonate as solvent (0.5 M), at room temperature for 18 h. Twenty products **5** were produced in 9–95% yield, some of which were calculated by ^1^H‐NMR (nuclear magnetic resonance). The authors reported a study on the green aspects of their methodology compared to the state of the art, remarking on their green potential. Additionally, the protocol was applied to *Se*‐phenyl benzeneselenosulfonate **6a**, furnishing the compound **7a** in 94% yield. Diallylether **3b** and diethyl diallylmalonate **3c** successfully reacted with **4a** to afford the respective products **8a** and **8b** in 95% and 84% yield, respectively. A gram‐scale reaction (5 mmol) was carried out to give the product **5a** in 80% yield. Finally, the methodology was used to produce (79% yield) a derivative of the API Apronal. It is noteworthy that the authors made a very thorough and detailed study of the reaction mechanism.

**SCHEME 3:**
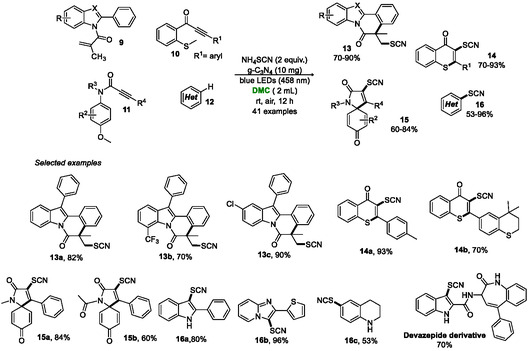
Synthesis of thiocyanated heterocycles **13**‐**16** [22].

Zeng et al. [22] developed a protocol to achievethiocyanated heterocyclesthrough a metal‐free photocatalytic reaction using NH_4_SCN (2 equiv.), under catalysis of graphitic carbon nitride (g‐C_3_N_4_, 10 mg) in dimethyl carbonate (Scheme 3). At room temperature, after 12 h, several substrates were investigated to afford thiocyanatedindolo/benzimidazo[2,1‐*a*]isoquinolin‐6(5*H*)‐ones **13**, thiocyanatedthioflavones **14**, thiocyanatedazaspiro[4.5]trienones **15**, and thiocyanated heterocycles **16** were furnished in good yields, from substrates **9**, **10**, **11**, and **12**, respectively. The synthesis of **13a** and **16a** was scaled up (from 0.2 to 5 mmol), and they were obtained in 60% and 71% yield, respectively. Moreover, a Devazepide derivative, which is commercialized as a cholecystokinin antagonist, was prepared in 70% yield after 76 h of reaction. Finally, the authors studied the sensitivity of their strategy and observed that the parameter that affected the reaction performance was the light intensity.

**SCHEME 4:**
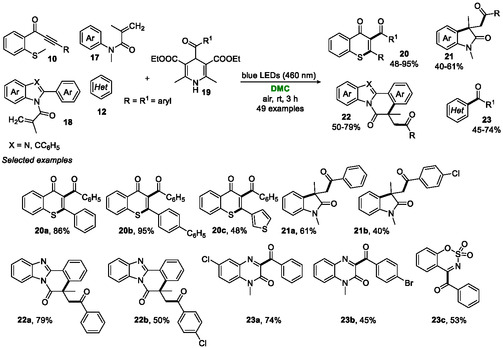
Blue light‐promoted synthesis of aroylated heterocycles **20**‐**23** [23].

In 2022, Chen, Zeng et al. [23] also proposed a blue light‐promoted strategy to prepare aroylated heterocycles with 4‐acyl‐1,4‐dihydropyridine **19** as acylating agent in dimethyl carbonate without any bases or additives. The best reaction conditions involve the irradiation of a mixture of a diversity of substrates with the reagent **19** (2 equiv.) with blue LEDs for 3 h. By this procedure, aroylatedthioflavones **20**, 3,3‐dialkyl 2‐oxindoles **21**, benzimidazo/indolo[2,1‐*a*]isoquinolin‐6(5*H*)‐ones **22**, quinoxaline‐2‐(1*H*)‐ones and benzo[*e*][1,2,3]oxathiazine‐2,2‐dioxides **23** were obtained in yields of 45–95% overall, starting from substrates **10**, **12**, **17**, and **18**, respectively (Scheme 4). The protocol was also suitable for the synthesis of drug and natural product derivatives, such as Ibuprofen, Flurbiprofen, Estrone, L‐Menthol, and Isoxepac derivatives in 65%, 50%, 55%, 56%, and 45% yield, respectively, after 12 h. Additionally, a gram‐scale reaction (4 mmol) was conducted, and the product **20a** was formed in 66% yield after 6 h. Moreover, the authors tested the reaction under solar irradiation, and after 6 h, the product **20a** was reached in 60% yield. Finally, a study on the sensitivity and reproducibility of the protocol was carried out, demonstrating that only the light intensity is responsible for the reaction success.

**SCHEME 5:**
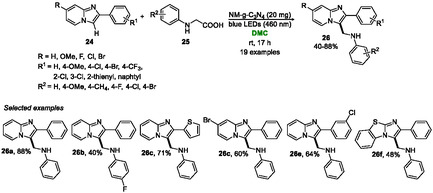
Blue LEDs irradiation promoted the decarboxylative coupling reaction [24].

In 2022, Shi et al. [24] published a photoinduced work on a decarboxylative coupling reaction of imidazo‐fused heterocycles **24** and *N*‐arylglycines **25**, catalyzed by carbon nitride nanosheets (NM‐g‐C_3_N_4_, 20 mg) previously prepared (Scheme 5). Nineteen products, **26** were accessed after 17 h at room temperature under blue LEDs irradiation in 40–88% yield. Various starting materials **24** (0.2 mmol) and **25** were suitable and demonstrated to react smoothly under the optimal reaction conditions. Moreover, a large amount (5 mmol) of **24a** and **25a** could be used, and the corresponding product **26a** was obtained in 77% yield. The recyclability of the catalyst was studied, and it could be recovered and reused seven times without loss of activity.

**SCHEME 6:**
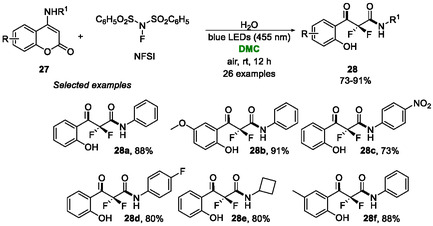
Synthesis of α,α‐difluoro‐β‐ketoamides **28** through photocatalytic multicomponent reaction [25].

In 2023, Li et al. [25] proposed a photocatalytic multicomponent reaction of 4‐aminocoumarins **27** (0.2 mmol), *N*‐fluorobenzenesulfonimide (NFSI, 2 equiv.), and water (5 equiv.) to prepare α,α‐difluoro‐β‐ketoamides **28** (Scheme 6). Several 4‐aryl‐ and 4‐alkylaminocoumarins **27** were employed, and under blue LEDs irradiation, after 12 h, twenty‐six products were formed in 73–91% yield. Except in the cases of the presence of halogens (fluorine, chlorine, and bromine) at position 6 of the 4‐aminocoumarin, in which only traces of the corresponding products were furnished. Finally, a scaled‐up reaction using 5 mmol of the substrate **27a** was tested, and the product **28a** was obtained in 82% yield.

**SCHEME 7:**
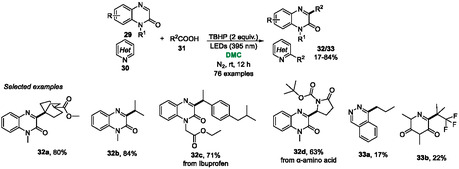
Decarboxylative C–H (amino)alkylation in DMC as solvent [26].

In 2023, Xu et al. [26] investigated the green decarboxylative C–H (amino)alkylation between an excess of carboxylic acid **31**(2 equiv.) and (amino)alkylating reagents **29** or **30** (0.2 mmol). The reaction proceeded in the absence of photocatalysts and metals, in the presence of 2 equiv. of *tert*‐butyl hydroperoxide as radical initiator and dimethyl carbonate as solvent, at room temperature, under nitrogen atmosphere for 12 h (Scheme 7). A very wide range of products **32** and **33** (seventy‐six examples) were synthesized in yields from 17% to 84% and among them, some compounds containing structures of pharmaceutical interest, derived from α‐, β‐, γ‐, and δ‐amino acids, and drugs such as Loxoprofen, Flurbiprofen, oleic acid, Gemfibrozil, and Ibuprofen. Moreover, product **32a** was achieved on a large scale (5 mmol) in 72% yield after 36 h of reaction. Another green aspect of this protocol is that the solvent could be recovered and reused for five cycles.

**SCHEME 8:**
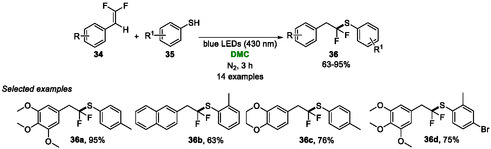
Synthesis of α,α‐difluoroalkylthioethers **36** promoted by blue LEDs irradiation [27].

In 2024, Zuo et al. [27] described a blue LEDs‐induced strategy to obtain α,α‐difluoroalkylthioethers **36** through a hydrothiolation of *gem*‐difluoroalkenes **34** with aryl thiols **35** (Scheme 8). Substrate **34** (0.1 mmol) reacted with **35** (1.5 equiv.) under a nitrogen atmosphere for 3 h, without a catalyst, and the targeted products **36** were achieved in 63–95% yield. The authors highlighted that the presence of two fluorines was essential for the reaction to occur. Unfortunately, a negative aspect of this work was that only aromatic thiols were suitable for the reaction.

**SCHEME 9:**
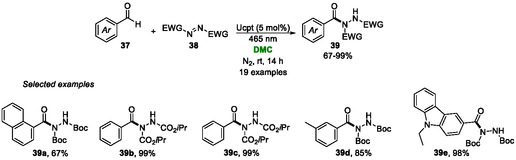
Uranyl‐organic framework as a catalyst in the photoinduced hydroacylation of dialkyl azodicarboxylates **38** [28].

In the same year, Chen et al. [28] developed the photoinduced hydroacylation of dialkyl azodicarboxylates **38** (0.2 mmol) with aldehydes **37** (3 equiv.). The reaction was catalyzed by uranyl‐organic framework (Ucpt, 5 mol%) in dimethyl carbonate under a nitrogen atmosphere at room temperature. A total of nineteen products **39**, were obtained after 14 h in 67–99% yield (Scheme 9).

**SCHEME 10:**
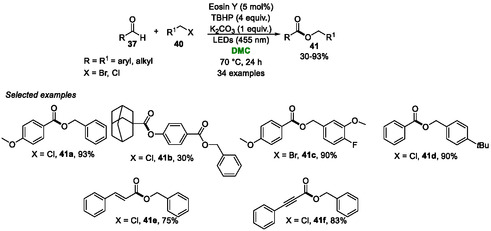
DMC as a solvent in the synthesis of nonsymmetrical esterified products **41** [29].

Still in 2024, Wang et al. [29] published another work on light irradiation in DMC. Under catalysis of Eosin Y (5 mol%), in the presence of K_2_CO_3_ (1 equiv.) and TBHP (4 equiv.), various aldehydes **37** and halides **40** reacted to furnish nonsymmetrical esterified products **41** in dimethyl carbonate, at 70°C after 24 h of reaction (Scheme 10). A total of thirty‐four esters were obtained starting from both aromatic and aliphatic halides and aldehydes, in 30–93% yield. Interesting, aldehydes bearing unsaturated bonds preserved it in the corresponding products.

**SCHEME 11:**
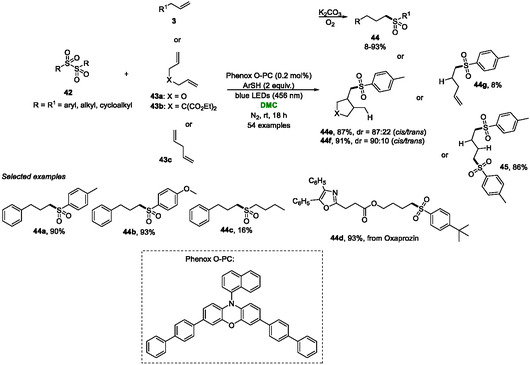
Hydrosulfonylation reaction promoted by blue LEDs irradiation in DMC [30].

In 2024, De Vos et al. [30] proposed blue light‐promoted methodology for the hydrosulfonylation of nonactivated alkenes **3** with disulfones **42**, affording the targeted products **44** in 8–93% yield. The best reaction conditions were found using 2 equiv. of both reagents, **3** and 2,4,6‐triisopropylthiophenol, in the presence of 0.2 mol% of the phenoxazine photocatalyst (Phenox‐O‐PC) in dimethyl carbonate, under a nitrogen atmosphere, at room temperature for 18 h. After that time, 2 equiv. of K_2_CO_3_ were added under an oxygen atmosphere for 2 h to form disulfide from the excess of thiophenol, which was almost quantitatively recovered (Scheme 11). The reaction worked well with a wide range of substrates **3** and **42**, including bioactive molecule derivatives, such as Apronal, Allylestrenol, Oxaproxin, Metaxalone, Biotin, and Ciprofibrate. Moreover, diallylether **43a**, diethyl diallylmalonate **43b**,and penta‐1,4‐diene **43c** were investigated: in the first two cases, the desired products **44e** and **44f** were formed in 87% and 91% yield, respectively. In the last case, however, a mixture of mono‐ and bis‐sulfonylated products, **44g** (8%) and **45** (86%), was obtained. A gram‐scale reaction was carried out (from 0.6 to 6 mmol), and the product **44a** was obtained in 91% yield. Additionally, the authors evaluated the green chemistry aspects of their work, demonstrating its sustainability.

**SCHEME 12:**
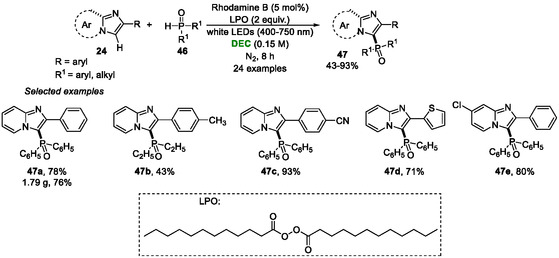
Synthesis of imidazo[1,2‐*a*]pyridines and 2‐phenylbenzo[*d*]imidazo[2,1‐*b*] thiazoles **47** [31].

In 2020, Gao et al. [31] employed diethyl carbonate as a green solvent in the phosphorylation reaction of imidazo[1,2‐*a*]pyridines **24** under visible light irradiation (white LEDs, 25 W). After optimization studies, the best reaction conditions were found using Rhodamine B (5 mol%) as photocatalyst, 2 equiv. of oxidant lauroyl peroxide (LPO), with an excess of phosphorus reagent **46** (2 equiv.), at room temperature under a nitrogen atmosphere, for 8 h (Scheme 12). Various imidazo[1,2‐*a*]pyridines **24**, bearing electron‐withdrawing and electron‐donating groups, or other aromatic substituents, as well as different phosphorus reagents **46**, were applied, demonstrating the applicability of the method. A total of twenty‐four imidazo[1,2‐*a*]pyridines and 2‐phenylbenzo[*d*]imidazo[2,1‐*b*] thiazoles **47** were obtained in moderate to excellent yields. The authors related that 2‐alkyl‐substituted imidazo[1,2‐*a*]pyridine and imidazo[1,2‐*a*]pyridine substrates did not react under the optimal conditions. In addition, a gram‐scale reaction (from 0.3 to 6 mmol) was carried out, reaching the product **47a** in 76% yield.

**SCHEME 13:**
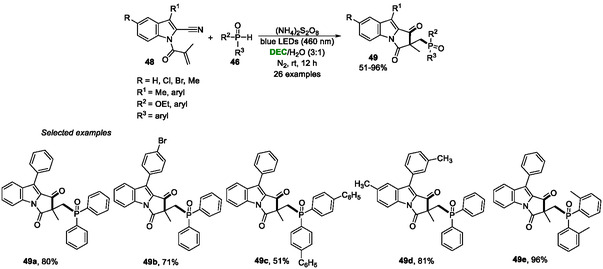
DEC/H_2_O solvent mixture in the synthesis of phosphorylated pyrrolo[1,2‐*a*]indolediones **49** [32].

In 2022, Huang et al. [32] described the synthesis of twenty‐six phosphorylated pyrrolo[1,2‐*a*]indolediones **49** in good to excellent yields. The authors investigated different solvents mixed with water, such as CH_3_CN, PEG‐400, 2‐MeTHF, DMC, and water alone. The best results were achieved using a mixture of diethyl carbonate and water as a solvent. The presence of water was necessary, as the authors highlighted in the last step of the reaction, which involved the hydrolysis to achieve the desired products. The reaction was promoted by visible light (blue LEDs, 10 W) under a nitrogen atmosphere, for 12 h at room temperature, using 2 equiv. of both phosphine oxides **46** and the oxidant (NH_4_)_2_S_2_O_8_, without metal or photocatalysts (Scheme 13). This protocol was suitable for various 1‐acryloyl‐2‐cyanoindoles **48** and different phosphine oxides **46**, bearing electron‐withdrawing and electron‐donating groups at the aromatic ring and indole moiety. Satisfactorily, a gram‐scale reaction was carried out using 3 mmol (instead of 0.2 mmol) of the starting material, and the compound **49a** was obtained in 73% yield.

**SCHEME 14:**
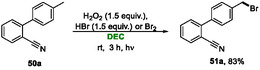
Synthesis of 4′‐(bromomethyl)‐2‐cyanobiphenyl **50a** in DEC as green solvent [33].

Sveegaard [33] proposed the synthesis of 4′‐(bromomethyl)‐2‐cyanobiphenyl **50a** in diethyl carbonate, replacing less greener solvents, such as carbon tetrachloride and chlorobenzene (Scheme 14). Compound **51a** is an important intermediate to access biologically active molecules, such as antihypertensive, antihyperglycemic, and anticancer agents. Compound **51a** was obtained in 83% yield after irradiation with visible light for 3 h at room temperature, in the presence of H_2_O_2_ (35 wt%) and HBr (1.5 equiv. each), instead of using toxic Br_2_ as a bromine source. The reaction was also tested in propylene carbonate and dimethyl carbonate, and product **51a** was formed in 25% and 55% yield, respectively, after 24 h at 40°C in the presence of 1 equiv. of both H_2_O_2_ and HBr. Moreover, a gram‐scale reaction was carried out, and the product was achieved in 96% yield, purified by a simple filtration.

## 3 OCs in Metal‐Catalyzed Reactions

### 3.1 Pd‐Catalyzed Reactions

**SCHEME 15:**
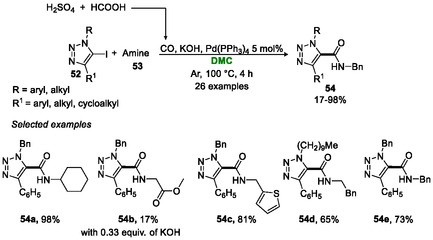
Pd‐catalyzed synthesis of 1,2,3‐triazole‐5‐carboxamides **54** [34].

In 2019, De Albuquerque et al. [34] described the synthesis of 1,2,3‐triazole‐5‐carboxamides **54** through the palladium‐catalyzed reaction between 5‐iodo‐1,2,3‐triazoles **52** and an excess of amines **53**. The desired products **54** were formed in good to excellent yields after 4 h of reaction at 100°C, in dimethyl carbonate as an eco‐friendly solvent, under argon atmosphere, in the presence of 5 mol% of Pd(PPh_3_)_4_, 1.5 equiv. of KOH and CO (Scheme 15). This last was produced ex situ by the reaction between an equimolar amount (4 equiv.) of H_2_SO_4_ and HCOOH, known as Morgan's reaction. In this study, various 5‐iodo‐1,2,3‐triazoles **52** and amines **53** were employed, and all were well tolerated. Exceptions were diethyl amine, piperidine, and aniline, which had proven to be nonreactive due to their low nucleophilicity and capacity to coordinate the palladium species.

**SCHEME 16:**
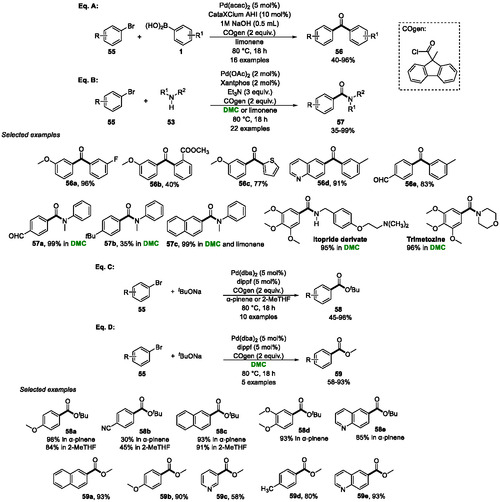
Carbonylative coupling of boronic acids and aryl bromides, aminocarbonylation, and alkoxycarbonylation in green solvents [35].

In 2020, Ismael et al. [35] developed a study on carbonylative coupling of boronic acids and aryl bromides, aminocarbonylation, and alkoxycarbonylation, investigating different green solvents. Limonene was found to be the best solvent for the carbonylative coupling of boronic acids **1** and aryl bromides **55** with CO generated in situ from COgen, 9‐methyl‐9*H‐*fluorene‐9‐carbonyl chloride, affording the desired products **56** in 40–96% yield (Scheme 16, Eq. A). By sightly changing the precedent protocol, the authors studied the aminocarbonylation of aryl bromides **55**, for which the best solvents were dimethyl carbonate and limonene, which could suitably displace the commonly used solvent toluene. The corresponding products **57** were reached in 35–99% yield after 18 h at 80°C, through a catalytic system of Pd(OAc)_2_ and 4,5‐bis(diphenylphosphino)−9,9‐dimethyl−xanthene (XantPhos) as ligand. Noteworthy, the commercial sedative Trimetozine and an Itopride derivative, used to treat gastrointestinal symptoms, were prepared in excellent yields (Scheme 16, Eq. B). Moreover, Pd‐catalyzed alkoxycarbonylation of aryl bromides **55** was investigated, and the better solvents of the reaction were α‐pinene and 2‐MeTHF, affording the products **58** in 45–98% yield (Scheme 16, Eq. C). Finally, dimethyl carbonate was studied in the alkoxycarbonylation reaction as a methylating agent to synthesize methoxycarbonylated compounds **59** (Scheme 16, Eq. D).

**SCHEME 17:**
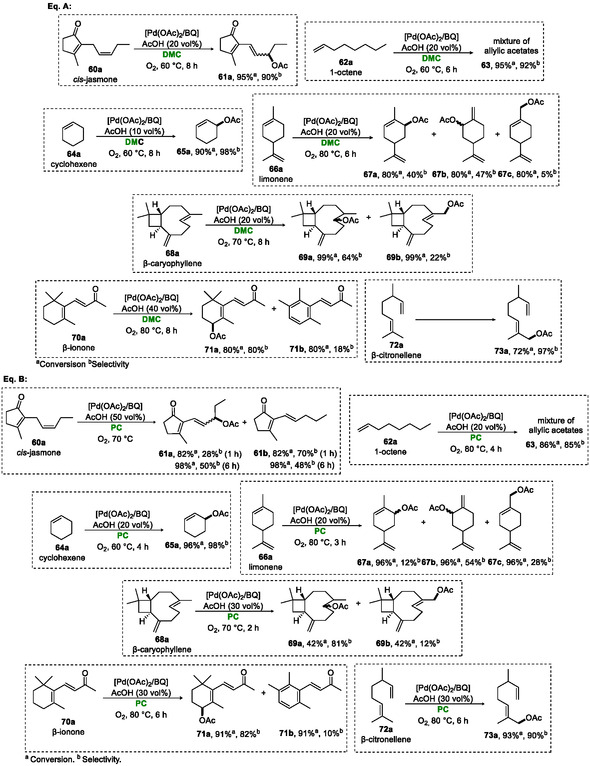
DMC and PC as green solvents in allylic oxidation [36].

In 2021, Costa et al. [36] tested green solvents, dimethyl carbonate (DMC), methyl isobutyl ketone (MIBK), and propylene carbonate (PC), as reaction media for the allylic oxidation of a series of substrates: α‐alkenes and alkenes containing internal disubstituted, trisubstituted, and tetrasubstituted C–C double bonds, including biomass‐based alkenes **60a**, **62a**, **64a**, **66a**, **68a**, **70a**, **72a** (Scheme 17). For the studies using DMC (Scheme 17, Eq. A) and PC (Scheme 17, Eq. B) as solvents, the authors used 0.20 M of alkenes, a mixture of Pd(OAc)_2_ (10 mM), *p*‐benzoquinone (BQ) (50 mM) as catalyst, gas phase O_2_ at 10 atm, and a specific amount of AcOH for each substrate, achieving the corresponding products **61a**, **63**, **65a**, **67a**‐**c**, **69a**,**b**, **71a**,**b**, **73a** in moderate to excellent yields. The oxidation reactions were shown to be efficient with alternative solvents in the presence of reduced amounts of AcOH, presenting high conversion and good selectivity, thus proving that the use of these solvents can be an excellent alternative to conventional ones. Furthermore, the authors highlight that under the same conditions, the reactions with all alkenes were faster in PC solutions than in DMC and MIBK.

**SCHEME 18:**
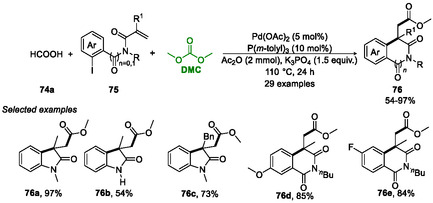
DMC as solvent and reagent in the synthesis of functionalized isoquinoline‐1,3‐diones and oxindoles **76** [37].

In 2022, Wen et al. [37] described the use of dimethyl carbonate both as a solvent and a reactant to prepare functionalized isoquinoline‐1,3‐diones and oxindoles **76** via palladium‐catalyzed intramolecular Heck/carbonylation from starting materials **75**. By reacting the substrates in the presence of formic acid **74a** as a CO source, 5 mol% of Pd(OAc)_2_ as a catalyst, 10 mol% of P(*m*‐tolyl)_3_ as a ligand, and 1.5 equiv. of K_3_PO_4_ as base, at 110°C for 24 h, it was possible to produce twenty‐nine compounds in good to excellent yields, showing a very good scope (Scheme 18).

**SCHEME 19:**
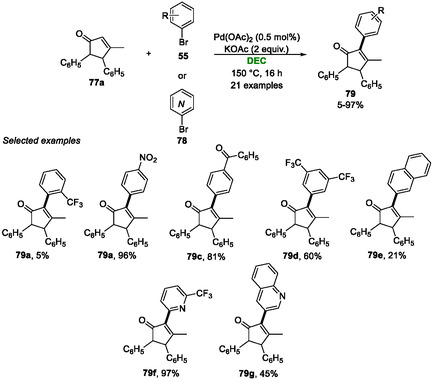
DEC as solvent in the arylation of antipyrine **77a** [38].

In 2020, Sasmal et al. [38] published a work on the arylation of antipyrine **77a** with aryl bromides **55**, also containing nitrogen**78**, catalyzed by Pd(OAc)_2_ (0.5 mol%) in the presence of KOAc (2 equiv.) as base in diethyl carbonate. Different substrates were investigated, and compounds **79** were obtained in yields of 5–97% by stirring at 150°C for 16 h (Scheme 19). Some starting materials did not undergo to reaction, such as 1‐bromo‐4‐(*tert*‐butyl)benzene, 4‐bromoanisole, and 1,2‐dibromobenzene; in these cases, dimethylacetamide was required as an alternative solvent.

**SCHEME 20:**
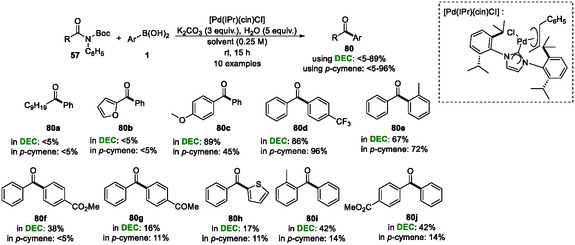
Green solvents in the Suzuki–Miyaura cross‐coupling reaction [39].

In 2021, Lei et al. [39] developed the first study on various green solvents applied for the Suzuky–Miyaura cross‐coupling of amides **57**, including diethyl carbonate and *p*‐cymene. The authors underlined the great importance of the possibility to use greener, safer, and healthier solvents than those usually used in this type of reaction, i.e., tetrahydrofuran, classified as a hazardous solvent. The reaction was conducted with 2 equiv. of aryl boronic acids **1**, in the presence of 3 mol% of Pd species, 3 equiv. of K_2_CO_3_, and 5 equiv. of H_2_O at 23°C for 15 h, to achieve the targeted products **80** (Scheme 20).

**SCHEME 21:**
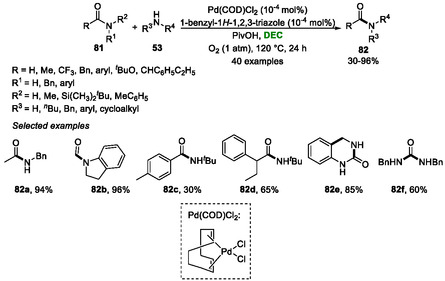
DEC as solvent in the transamidation of primary, secondary, and tertiary amides **82** and ureas with amines **53** [40].

In 2023, Urgoitia et al. [40] published the transamidation of primary, secondary, and tertiary amides **81**, and ureas with amines **53** (Scheme 21). The reaction was promoted by molecular oxygen (1 atm), in the presence of palladium, using the biodegradable and eco‐friendly solvent diethyl carbonate. The methodology tolerated a wide range of substrates, and forty amides **82** were produced in 30–96% yield, after 24 h of reaction at 120°C. The authors used 2 equiv. of amine **53** and 3.6 equiv. of pivalic acid (PivOH) as a proton shuttle, and all of them contain 0.1 ppm of palladium impurities. A gram‐scale reaction was examined (34 mmol of starting material instead of 0.54 mmol), and compound **82a** was achieved in 70% yield.

**SCHEME 22:**
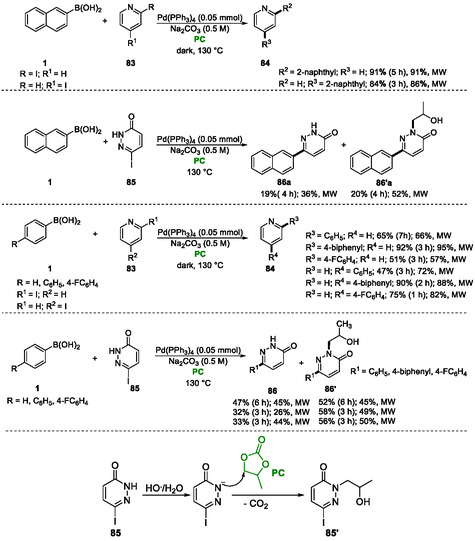
PC as agreen solvent in the cross‐coupling Suzuki–Miyaura reaction [41].

In 2019, Czompa et al. [41] developed a study on the use of propylene carbonate as a green solvent in the cross‐coupling Suzuki–Miyaura reaction. The reaction was carried out both using conventional heating and under microwave irradiation (MW). The starting materials used were heterocyclic substrates **83**, such as 2‐iodopyridine, 4‐iodopyridine, and 6‐iodopyridazin‐3(2*H*)‐one **85** (1 mmol) in reactions with boronic acids **1**: 2‐naphthylboronic, phenylboronic, 4‐biphenylboronic, and 4‐fluorobenzeneboronic acids (1.25 equiv.). In a typical procedure, the reagents are stirred in the presence of 0.05 mmol Pd(PPh_3_)_4_ as catalyst and 2 mL of Na_2_CO_3_ 0.5 mol/L as base in PC at 130°C. When 2‐iodopyridine and 4‐iodopyridine were used, the reaction was conducted in the dark, as they are light and air‐sensitive (Scheme 22). The reaction time under microwave irradiation was 1 h, whereas under conventional heating, it was between 1 and 7 h, depending on the substrates employed. In general, the yields obtained under MW irradiation were higher than those under conventional heating, perhaps due to greater temperature homogeneity. Sixteen products **84** and **86** were obtained in 19–95% yield (conventional heating) and in 26–97% (under MW irradiation). Unfortunately, the authors commented that the PC is responsible for the formation of side products **86′**, in the cases of the reactions between 6‐iodopyridazin‐3(2*H*)‐one **85** with all boronic acids, due to the deprotonation of the pyridazinone **85** and the opening of PC, promoted by the base, forming the intermediate **85′**.

**SCHEME 23:**
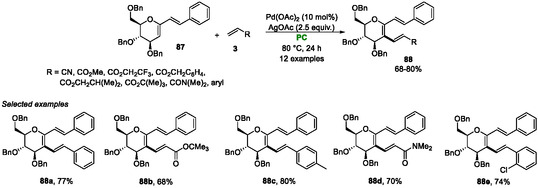
PC as green solvent in the synthesis of 1,2‐disubstituted glucals **88** [42].

In 2024, Maikhuri et al. [42] described the preparation of 1,2‐disubstituted glucals **88** in propylene carbonate as a solvent (Scheme 23). The method involves stirring a mixture of C1‐styrylglucal **87** and alkenes **3** in the presence of Pd(OAc)_2_ (5 mol%) as catalyst and AgOAc (2.5 equiv.) as oxidant. Various alkenes were investigated, such as styrenes, acrylates, acrylamide, and acrylonitrile, and the products **88** were obtained satisfactorily in 68–80% yield. A gram‐scale reaction was conducted, and the product **88a** was prepared in 74% yield. After the preparation of this class of compounds, they were employed in the 6π‐electrocyclization aromatization, in ethylene glycol at 180°C for 3 h, to access 2*H*‐chromenes.

### 3.2 Fe‐Catalyzed Reactions

**SCHEME 24:**
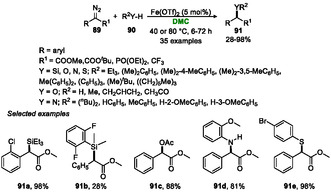
Dimethyl carbonate as a solvent in the synthesis of products **91** [43].

In 2019, Tanbouza et al. [43] proposed the replacement of chlorinated solvents with dimethyl carbonate in the reaction of insertion of α‐diazocarbonyl compounds **89** in Si=H, S=H, N=H, and O=H bonds of compounds **90** (Scheme 24). The reaction was catalyzed by iron species, Fe(OTf)_2_ (5 mol%), at 40°C or 80°C after 6–72 h, and the desired products **91** were achieved in 28–98% yield. A wide range of substrates was tolerated under the reaction conditions, including α‐diazocarbonyl compounds with electron‐donating and electron‐withdrawing groups, various silanes, aryl thiols, primary and secondary amines, acetic acid, and alcohols.

**SCHEME 25:**
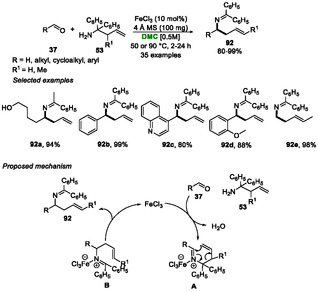
Synthesis of α‐substituted homoallylamines **92** [44].

Gadde et al. [44] developed the first methodology to access α‐substituted homoallylamines **92** starting from equimolar amounts of aldehydes **37** and 1,1‐diphenylhomoallylamines **53** (Scheme 25). A wide range of substrates was investigated, and the products were obtained in 80–98% yield after 2–24 h, at 50 °C or 90°C, in the presence of FeCl_3_ as Lewis acid catalyst, demonstrating the good applicability and generality of the reaction. Dimethyl carbonate was used as a solvent, except for the product **92a**, which was obtained in propylene carbonate. Additionally, a gram‐scale (5 mmol) reaction was carried out, and after 9 h, the product **92b** was furnished in 94% yield. Moreover, the authors proposed a plausible reaction mechanism, in which, first, the iminium intermediate **A** is formed, which then undergoes a cationic 2‐aza‐Cope rearrangement to reach species **B**. This last generates the desired product **92** and regenerates the catalyst.

**SCHEME 26:**
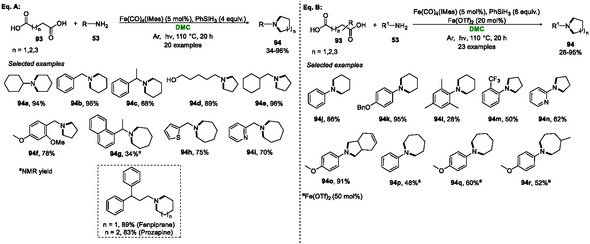
Iron‐catalyzed one‐pot hydrosilylation to achieve *N*‐substituted cyclic amines **94** [45].

Wei et al. [45] described for the first time a chemoselective synthesis of the *N*‐substituted cyclic amines **94** (piperidines, pyrrolidines, and azepanes) via iron‐catalyzed one‐pot hydrosilylation starting from dicarboxylic acids **93** and amines **53**, with hydrosilanes and dimethyl carbonate as solvent (Scheme 26). This procedure had two optimal reaction conditions. First, the hydrosilylation of diacids in the presence of aliphatic primary amines was done using 0.5 mmol of dicarboxylic acids **93** and 0.5 mmol of amines **53** in the presence of phenylsilane (PhSiH_3_) as the hydride source and 5 mol% of iron(0) *N*‐heterocyclic carbene complex [Fe(CO)_4_(IMes)] in DMC as solvent, under visible light irradiation (24 W compact fluorescent lamp) at 110°C during 20 h under argon atmosphere. Then, through the hydrolysis with THF/NaOH 2 mol/L, the desired products were formed in 34–96% yield (Scheme 26, Eq. A). On the other hand, the hydrosilylation of diacids in the presence of primary arylamines **53** used 6 equiv. of PhSiH_3_ and 20 mol% of the Lewis acids Fe(OTf)_2_ as additive, furnished twenty‐three examples of **94** in 28–95% yield (Scheme 26, Eq. B). This methodology tolerated the preparation of a wide range of *N*‐alkylated and *N*‐arylated cyclic amine derivatives in moderate to excellent yields, starting from glutaric, succinic, and adipic acids, yielding piperidines, pyrrolidines, and azepanes, respectively. Furthermore, the authors demonstrated the synthetic utility of this method in the preparation of drug molecules Fenpiprane and Prozapine.

**SCHEME 27:**
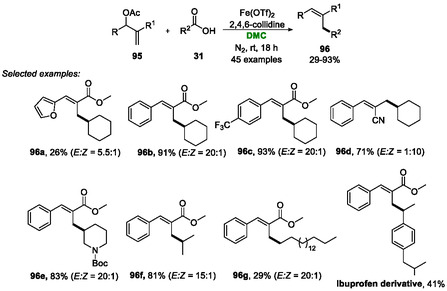
DMC as solvent in the alkylation of MBH acetates **96** [46].

In 2024, Golagani et al. [46] published a study to obtain products of alkylation of Morita–Baylis–Hilman (MBH) acetates, using dimethyl carbonate as a green solvent. The best reaction conditions involved using 0.3 mmol of MBH acetate **95**, 0.6 mmol of carboxylic acids **31**, 5 mol% of Fe(OTf)_2_ as catalyst, 20 mol% of 2,4,6‐collidine as base, and 3 mL of dimethyl carbonate as solvent, at room temperature, under irradiation with blue LEDs (40 W, 457 nm) for 18 h (Scheme 27). Under these conditions, the products were obtained as an *E*/*Z* mixture of isomers (*E*:*Z* ratio = 20:1). The authors employed methyl acrylate derivatives with electron‐withdrawing and donating substituents, affording good to excellent yields of the expected products **96**. Furthermore, derivatives of a wide range of aldehydes, alkyl ethers, and complex molecules could be prepared in a range of 29–93% yield. When MBH acetate was used with a nitrile group, the major isomer was *Z* (**96d**). The authors suggested that this was due to the high radical‐stabilization energy of the *Z*‐allylic radical originating from MBH acetate. Additionally, the authors employed the methodology to obtain different molecules with pharmacological activity, such as Ibuprofen.

**SCHEME 28:**
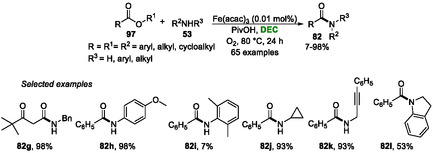
Synthesis of secondary and tertiary amides **82** [47].

In 2024, Obieta et al. [47] developed a new methodology to access secondary and tertiary amides **82** from various amines **53** and carboxylic esters and acids **97** under iron catalysis in the presence of oxygen. The best reaction conditions were found using 0.54 mmol of esters or acids **97**, an excess of amine **53** (2 equiv.), and an equimolar amount of pivalic acid as an additive in the presence of 0.01 mol% of Fe(acac)_3_, in DEC under an oxygen atmosphere for 24 h at 100°C (Scheme 28). In this reaction, it was demonstrated for the first time that oxygen plays a role in inducing amidation. A large variety of amines **53** and esters/acids **97** were subjected to the optimal reaction conditions, thus sixty‐five products **82** were reached in 7–98% yield. When optically active substrates were used, a retention of configuration was observed. Moreover, a gram‐scale reaction was performed between phenyl benzoate (10 mmol) and benzamide, yielding the corresponding productin 95% yield.

**SCHEME 29:**
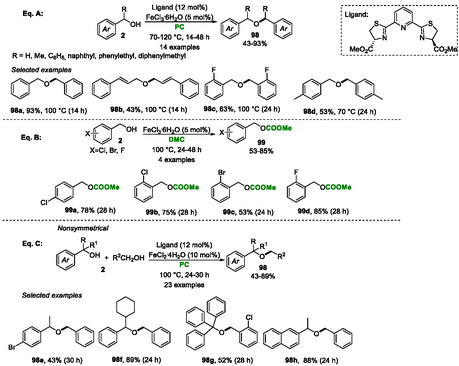
Synthesis of symmetrical and unsymmetrical ethers **98** (Eqs. A and C), and methyl carbonates **99** (Eq. B) [48].

In 2023, Slimi et al. [48] proposed two strategies, one for symmetric and the other for nonsymmetric etherification of benzyl alcohols **2**. In the first case, the reaction was catalyzed by 5 mol% of FeCl_3_ · 6H_2_O, while 10 mol% of FeCl_2_·4H_2_O was used as a catalyst to prepare the nonsymmetrical derivatives. In both cases, 12 mol% of pyridine bis‐thiazolinewas used as ligand, with propylene carbonate as green solvent (Scheme 29, Eqs. A and C). The symmetrical ethers **98** were formed in 43–93% yield after 14–48 h of reaction at 70–120°C. The authors also carried out the reaction in dimethyl carbonate, and they observed that, unexpectedly, the benzyl alcohol substituted with halogen groups, such as chlorine, bromine, and fluorine, at different positions, did not furnish the symmetrical ethers, because they reacted with DMC, forming the methyl carbonates **99** (Scheme 29, Eq. B). The nonsymmetrical ethers, in contrast, were achieved in 43–89% yield after 24–30 h of reaction at 100°C. The substrates 1(1‐(benzyloxy)ethyl)‐4‐(trifluoromethyl)benzene and 1‐(1‐(benzyloxy)ethyl)‐3‐(trifluoromethyl)benzene were not suitable substrates to the cross‐etherification.

### 3.3 Cu‐Catalyzed Reactions

**SCHEME 30:**
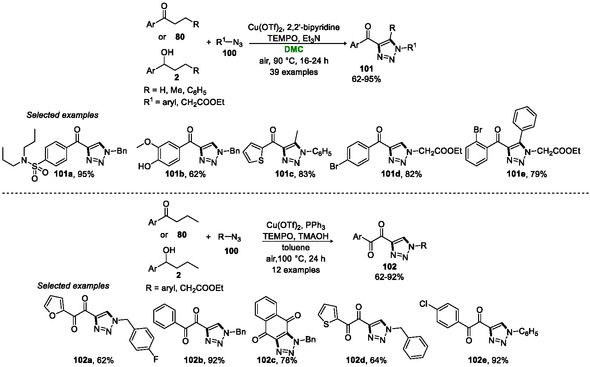
Tandem multiple oxidative dehydrogenation and cycloaddition to prepare 4‐acyl‐1,2,3‐triazoles and 4‐diketo‐1,2,3‐triazoles **101** and 4‐diketone‐1,2,3‐triazoles **102** [49].

In 2022, Huang et al. [49] proposed a strategy to obtain 4‐acyl‐1,2,3‐triazoles and 4‐diketo‐1,2,3‐triazoles **101** through a tandem multiple oxidative dehydrogenation and cycloaddition promoted by Cu/TEMPO (Scheme 30). Different aryl‐alkylketones **80** or alcohols **2** (1 mmol) were investigated in reactions with various azides **100** (1.5 equiv.), in the presence of Cu(OTf)_2_ (10 mol%) as catalyst, 2,2′‐bipyridine (0.2 equiv.) as ligand, TEMPO (0.3 equiv.) as co‐catalyst, and Et_3_N (0.4 equiv.) as base in dimethyl carbonate at 90°C for 16–24 h. In this way, it was possible to prepare thirty‐nine targeted products **101** in 62–95% yield. The authors highlighted that when different butanone or butanol derivatives were reacted with azides (1.5 equiv.) in the presence of Cu(OTf)_2_ (10 mol%), PPh_3_ (0.2 equiv.), TEMPO (0.3 equiv.), and tetramethylammonium hydroxide (TMAOH, 0.25 equiv., 25% solution in MeOH), in toluene at 100°C, twelve examples of 4‐diketone‐1,2,3‐triazoles **102** were prepared in 62–92% yield.

**SCHEME 31:**
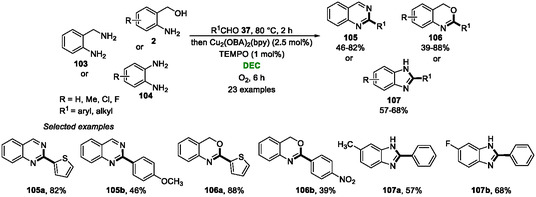
Synthesis of five‐or six‐membered *N*,*N*/*N*,*O* heterocycles **105**‐**107**in DEC as solvent [50].

In 2019, Tran et al. [50] described for the first time the use of diethyl carbonate and solid reusable catalyst in the synthesis of five‐ or six‐membered*N*,*N*/*N*,*O* heterocycles **105**, **106**, and **107**. The optimal reaction conditions were encountered using an equimolar amount of 2‐aminobenzyl amine **104** and aldehydes **37** (0.2 mmol) at 80°C for 2 h (Scheme 31). Then, 2.5 mol% of catalyst Cu_2_(OBA)_2_(bpy), a copper amine complex, and 1 mol% of TEMPO were added, and the resulting mixture was stirred for an additional 6 h under an oxygen atmosphere in diethyl carbonate as solvent. Through the coupling reaction with various aldehydes **37**, it was possible to prepare twelve desired products **105**, **106**, and **107** in 65–81% yield. Moreover, under the same reaction conditions, 2‐aminobenzyl alcohols **2** or 1,2‐phenylene diamines **103** were investigated as starting materials in the reaction with different aldehydes **37**. The catalyst Cu_2_(OBA)_2_(bpy) could be recovered and reused five times, maintaining the same performance.

### 3.4 Co‐Catalyzed Reactions

**SCHEME 32:**
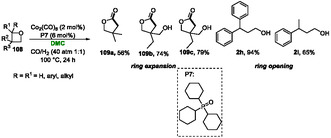
Cobalt species‐catalyzed ring expansion and reductive ring opening reaction of oxetanes **108** [51].

In 2022, Delolo et al. [51] developed a methodology for ring expansion and reductive ring opening reaction of oxetanes **108** (1 mmol), catalyzed by Co_2_(CO)_8_ and promoted by tricyclohexylphosphine oxide (P7) at 100°C for 24 h. Depending on the starting material structure, two pathways are possible: ring expansion or ring opening, thus achieving γ‐lactones **109** or alcohols **2** (Scheme 32). The authors reported that both P7 and syngas are vital for the reaction's success, driving the catalytic species to promote the carbonylation or the reduction. The ring expansion took place when there were no stable substituents at the oxetane, while ring opening occurred when the oxetane contained phenyl groups.

**SCHEME 33:**
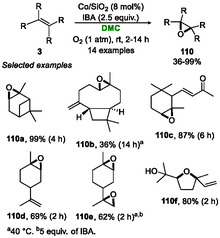
DMC as a solvent in the epoxidation of alkenes **3** [52].

In 2022, Almeida et al. [52] described the epoxidation of alkenes **3** in the presence of molecular oxygen as a final oxidant, *iso*‐butyraldehyde (IBA) as a sacrificial reagent, and a silica‐supported cobalt as a heterogeneous catalyst (calcinated at 550°C) (Scheme 33). Fourteen examples were obtained using 0.5 mmol of alkene **3**, IBA (2.5 equiv.), and the catalyst (8 mol%) under an O_2_ atmosphere in DMC as a solvent. A total of fourteen epoxides **110** were obtained in 36–99% yield under mild conditions, starting from bio‐renewable terpenes with different patterns of substitution at the C–C double bonds. Reaction times and all yields were determined by GC‐conversion analyses. The authors performed recycling experiments to evaluate the catalyst stability, and the results obtained through GC evidenced that even after 5 reuse cycles, the conversion rate remained the same, and the selectivity dropped from 79% to 72%. These results, together with others, such as inductively coupled plasma‐optical emission spectroscopy and transmission electron microscopy analyses, demonstrated the catalytic efficiency and stability under the optimal reaction conditions using DMC as the reaction medium.

### 3.5 W‐Catalyzed Reactions

**SCHEME 34:**
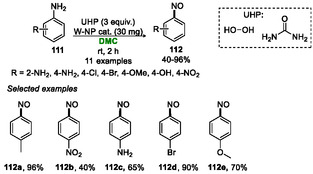
Synthesis of nitroso compounds **112** in DMC as solvent [53].

Nejad et al. [53] developed a methodology to prepare nitroso arenes **112** in good to excellent yields at room temperature for 2 h, using sodium tungstate‐supported magnetite nanoparticles as a catalyst (W‐NP cat, Scheme 34). The authors prepared and characterized the catalyst and employed it in the reaction with anilines **111** (1 mmol) and hydrogen peroxide/urea (UHP, 3 equiv.) as oxidant. Dimethyl carbonate was employed as a solvent, and its role in the reaction was to provide the final activation of UHP. Eleven products **112** were prepared in 40–96% yield. Among the tested anilines, only 2,4‐dinitro‐aniline could not be converted into the corresponding product. Moreover, the catalyst was recycled and reused six times, maintaining the protocol's efficiency.

**SCHEME 35:**
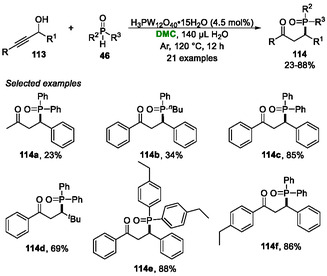
Preparation of γ‐ketophosphine oxides **114** in DMC as solvent [54].

In 2024, Yang et al. [54] developed the use of phosphotungstic acid (HPWA) with a Keggin structure as a catalyst for the phosphinylation of secondary propargyl alcohols **113** with phosphine oxides **46**. The products, γ‐ketophosphine oxides **114**, were obtained with good selectivity using dimethyl carbonate as an eco‐friendly solvent (Scheme 35). The optimal conditions involve stirring a mixture of 0.6 mmol of **113** and 0.3 mmol of **46** in the presence of 4.5 mol% of the catalyst HPWA for 12 h at 120°C in dimethyl carbonate, with 140 µL of H_2_O as an additive, under an argon atmosphere. The authors commented that adding water was important because it facilitated the reaction and increased the yield, acting in the reaction mechanism. By this procedure, twenty‐one examples of compounds **114** were prepared in 23–86% yield using different propargyl alcohols and various phosphine oxides. Various substituents at the propargylic alcohols were suitable for the reaction, except when the pendant group at the triple bond was a methyl, which gave the product **114a** in only 23% yield. Moreover, the phosphine oxide bearing an aliphatic group provided the respective product **114b** in 34% yield. Additionally, a gram‐scale (6 mmol) reaction was carried out under the optimal reaction conditions, and the product **114c** was furnished in 63% yield.

**SCHEME 36:**
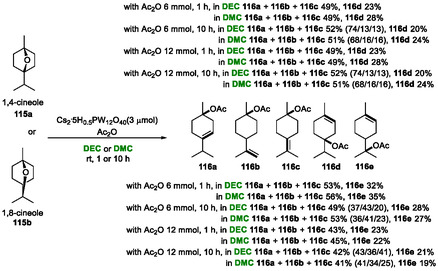
DMC and DEC as solvents in thepreparation of monoterpenic esters **116** [55].

In 2024, Lataliza‐Carvalho et al. [55] proposed a new protocol to obtain monoterpenic esters **116**, which may have flavoring properties (Scheme 36). The reaction was tested in various green solvents, such as methyl isobutyl ketone, methyl ethyl ketone, dimethyl carbonate (DMC), and diethyl carbonate (DEC). Following, we summarized the results obtained with DMC and DEC. The desired products were formed through the reaction between 1,4‐cineole **115a** or 1,8‐cineole **115b** under catalysis of acetic anhydride in the presence of acidic cesium salt of phosphotungstic acid, Cs_2.5_H_0.5_PW_12_O_40_ (CsPW) at 25°C. The authors studied two reaction times: 1 and 10 h, and two amounts of Ac_2_O: 6 and 12 mmol. From 1,4‐cineole, after 1 h of reaction, with 6 mmol of Ac_2_O, the conversions were 51% and 47% in DMC and in DEC, respectively. The selectivities to products **116a**, **116b**, and **116c** were 49% in both DMC and DEC; for product **116d**, they were 28% and 23% in DMC and in DEC, respectively. Increasing the amount of Ac_2_O to 12 mmol, the conversions after 1 h were 50% and 47% in DMC and DEC, respectively; the selectivities to **116a**, **116b**, and **116c** were 44% and 41% in DMC and DEC, respectively. For product **116d**, they were 19% and 22% in DMC and in DEC, respectively. Instead, after 10 h of reaction, with 6 mmol of Ac_2_O, the conversions were 69% and 67% in DMC and DEC, respectively. The selectivities to **116a**, **116b**, and **116c** were 51% (68/16/16) and 52% (74/13/13) in DMC and DEC, respectively, while for **116d**, they were 24% and 20% in DMC and DEC, respectively. With 12 mmol of Ac_2_O, after 10 h, the conversions were 86% and 77% in DMC and DEC, respectively; the selectivities to **116a**, **116b**, and **116c** were 45% (70/15/15) and 42% (72/14/14) in DMC and DEC, respectively. For product **116d**, they were 16% and 17% in DMC and in DEC, respectively. For 1,8‐cineole **116b**, in contrast, after 1 h of reaction with 6 mmol of Ac_2_O, the conversions were 68% and 81% in DMC and in DEC, respectively. The selectivities to products **116a**, **116b**, and **116c** were 56% and 53% in both DMC and DEC, respectively. For product **116e**, they were 25% and 32% in DMC and in DEC, respectively. Increasing the amount of Ac_2_O to 12 mmol, the conversions after 1 h were 81% and 83% in DMC and DEC, respectively; the selectivities to **116a**, **116b**, and **116c** were 45% and 43% in DMC and DEC, respectively. For product **116e**, they were 22% and 23% in DMC and in DEC, respectively. Instead, after 10 h of reaction, with 6 mmol of Ac_2_O, the conversions were 81% and 90% for DMC and DEC, respectively, and the selectivities to **116a**, **116b**, and **116c** were 53% (36/41/23) and 49% (37/43/20) in DMC and DEC, respectively. For product **116e** they were 27% and 28% in DMC and DEC, respectively. With 12 mmol of Ac_2_O, after 10 h, the conversions were 84% and 90% in DMC and DEC, respectively; the selectivities to **116a**, **116b**, and **116c** were 41% (41/34/25) and 42% (43/36/41) in DMC and DEC, respectively. For product **116e** they were 19% and 21% in DMC and in DEC, respectively. Additionally, the catalyst CsPW was stable and could be recovered and reused three times with no loss of performance.

### 3.6 Other Metals as Catalysts

**SCHEME 37:**
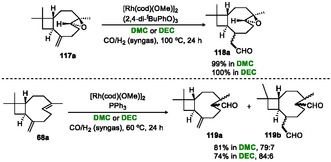
DMC and DEC as solvents in thehydroformylation of the sesquiterpenes caryophyllene oxide **117a** and β‐caryophyllene **68a** [56].

Faria et al. [56] in 2020, a study was reported on the use of different eco‐friendly solvents to promote the hydroformylation of the sesquiterpenes caryophyllene oxide **117a** and β‐caryophyllene **68a**. The authors evaluated a series of solvents, including dimethyl and diethyl carbonate. We emphasize that this work was previously addressed in a review article by our research group on the use of biobased solvents, such as 2‐MeTHF and *p*‐cyrene [5]. For caryophyllene oxide (**117a**, 4 mmol), the reaction was carried out using [Rh(cod)(OMe)]_2_ (5 μmol) as catalyst and (2,4‐di‐^*t*^BuPhO)_3_P (10 μmol) as ligand, in 20 mL of DMC or DEC, under 80 atm of syngas (CO/H_2_) at 100°C for 24 h, furnishing the corresponding product **118a** (Scheme 37). GC–MS analyses indicated 99% conversion when dimethyl carbonate was used and 100% for diethyl carbonate. For β‐caryophyllene (**68a**, 4 mmol), PPh_3_ replaced (2,4‐di‐^*t*^BuPhO)_3_P as ligand, and the reaction was performed at 60°C. The substrate **68a** reached 81% conversion when DMC was used, affording a mixture of mono‐hydroformylated (**119a**) and di‐hydroformylated (**119b**) products in a 79:7 ratio. When DEC was used, the mixture of products **119a** and **119b** was obtained with 74% of conversion and a **119a**:**119b** ratio of 84:6.

**SCHEME 38:**
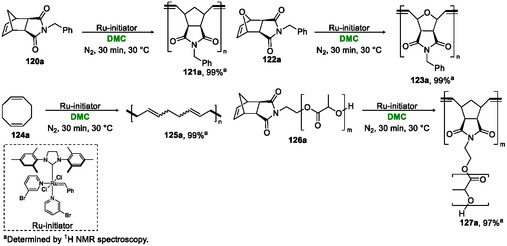
ROMPs [57].

Shin et al. [57] reported a ruthenium alkylidene mediated ring‐opening metathesis polymerization (ROMP), where they performed a comparison between conventional (tetrahydrofuran and chloroform) and green solvents (2‐methyl tetrahydrofuran, ethyl acetate, dimethyl carbonate, and acetone). They carried out the polymerization of norbornene **120a** (0.040 mmol), oxa‐norbornene **122a** (0.039 mmol), cyclooctadiene **124a** (0.094 mmol), and norbornenyl macromonomer **126a** (3.9 μmmol) in the presence of a ruthenium initiator and the selected solvent during 30 min at 30°C under nitrogen atmosphere, forming the corresponding products **121a**, **123a**, **125a**, **127a** (Scheme 38). The authors reported a good conversion, which was determined by ^1^H NMR, for the polymerization reactions using green solvents compared to conventional ones. Furthermore, they performed characterization analyses, such as gel permeation chromatography, to determine dispersivity (*Ð*). The use of these solvents provided promising results, as the values approached those obtained when polymerization was carried out in traditional solvents. Among the tested solvents, the results showed that DMC had the greatest potential applicability (Scheme 38).

**SCHEME 39:**
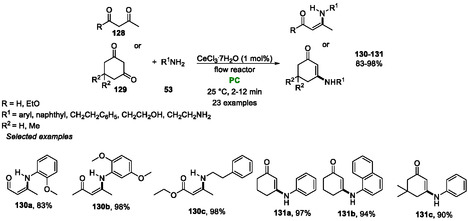
Flow reactor system in the synthesis of enaminones **130**‐**131** [58].

In 2023, Ramezani and Godarzi [58] described a flow procedure to obtain enaminones **130**‐**131** catalyzed by CeCl_3_^.^·7H_2_O (1 mol%) in propylene carbonate, starting from different β‐dicarbonyls **128**or 1,3‐cyclohexanediones **129**, and amines **53** (Scheme 39). Furthermore, the authors studied the same reaction in a conventional batch system at room temperature, under stirring. They compared the results, demonstrating the same efficiency with the advantage that the flow reaction was much faster: 2–12 min versus 1–3 h. The targeted products **130**‐**131** were synthesized in 83–98% yield, continuously collected, and then purified by simple filtration. The catalyst and solvent could be recovered and reused in the flow system up to six times without loss of efficiency.

## 4 OCs in Metal‐Free Catalysis

**SCHEME 40:**
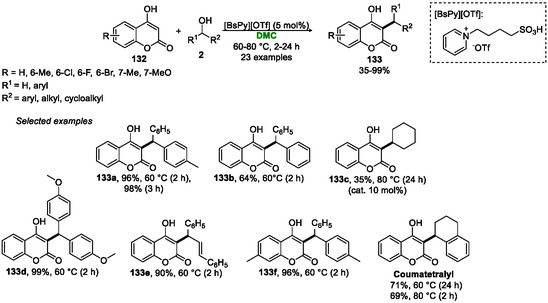
Ionic liquid‐catalyzed synthesis of 4‐hydroxycoumarin derivatives **133** [59].

In 2021, Li et al. [59] proposed a metal‐free pyridine‐based ionic liquid‐catalyzed synthesis of 4‐hydroxycoumarin derivatives **133** in dimethyl carbonate. The best reaction conditions were encountered when a mixture of **132** and alcohols **2** (1.2 equiv.) was used in the presence of 5 mol% of ionic liquid [BsPy][OTf] as catalyst, in DMC, at 60–80°C, in 2–24 h (Scheme 40). Various secondary alcohols **2**, bearing aromatic substituents with electron‐donating and electron‐withdrawing groups, as well as aliphatic alcohols **2** were employed, and all of them reacted smoothly. Moreover, hydroxycoumarin derivatives **132** were investigated, furnishing very good results. A gram‐scale reaction was carried out(5 mmol), and the product **133a** was obtained in 98% yield after 3 h of reaction. The protocol was also applied to the synthesis of the commercial rodenticide Coumatetralyl, which was obtained in 71% yield. Additionally, the authors investigated the recyclability of the ionic liquid, and they demonstrated that it could be recycled and reused four times.

**SCHEME 41:**
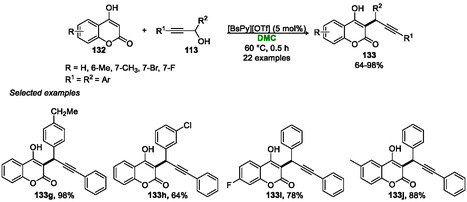
Sulfonic acid‐functionalized ionic liquid‐catalyzed preparation of C3‐substituted 4‐hydroxycoumarins **133** [45].

The following year, the same group [45] described another methodology to access C3‐substituted 4‐hydroxycoumarins **133**, now using pirydinium sulfonic acid‐functionalized ionic liquid [BsPy][OTf] as a catalyst. A variety of secondary aromatic propargylic alcohols **113** (0.5 mmol) reacted with 4‐hydroxycoumarin derivatives **132** (1.2 equiv.) in dimethyl carbonate at 60°C for 30 min, in the presence of 5 mol% of [BsPy][OTf], forming the targeted products **133** in 64–98% yield (Scheme 41). A gram‐scale reaction was carried out (6 mmol), and the product **133h** was formed in 90% yield under the same reaction conditions. Furthermore, the catalyst could be easily recovered and reused seven times without loss of efficiency.

**SCHEME 42:**
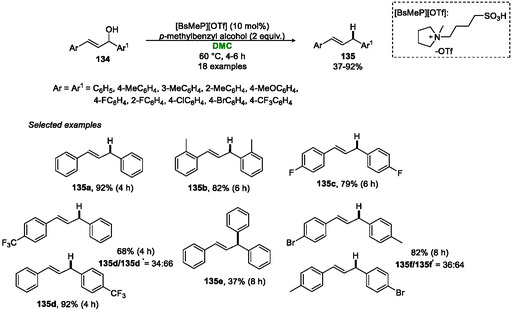
Pyrrolidine‐based IL in thereduction of aromatic allylic alcohols **134** [60].

In 2022, Liu et al. [60] developed a metal‐free protocol, in which the green solvent dimethyl carbonate acted as a stabilizer for the intermediates of the reduction of aromatic allylic alcohols **134** catalyzed by acidic pyrrolidine‐based ionic liquids (IL). The reaction occurred at 60°C for 4–6 h, in the presence of 10 mol% of the catalyst IL and 2 equiv. of *p*‐methylbenzyl alcohol as the reducing agent, to give the products **135** in 37–92% yield (Scheme 42). Several allylic alcohols **134** were investigated, bearing identical or different aryl groups at the 1 and 3 positions. In this last case, a mixture of products was obtained, depending on the position of the double bond. It was observed that the double bonds preferred to form near the electron‐rich aryl group. The authors related that reaction did not work with aliphatic allylic alcohols. A gram‐scale reaction (from 0.5–10 mmol) was performed, and the product **135a** was produced in 87% yield after 4 h. Additionally, the catalyst IL could be recycled and reused five times, maintaining the same performance.

**SCHEME 43:**
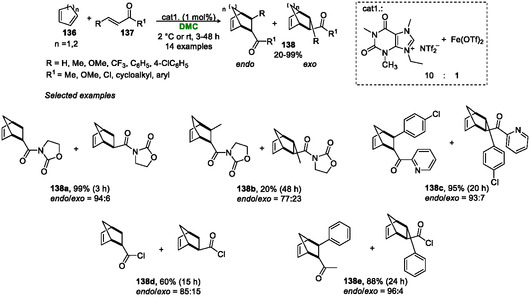
Caffeine‐derived salt as a catalyst in the Diels–Alder reaction [61].

Meng et al. [61] described the reaction between cyclic dienes **136** and dienophiles **137** using an iron(II)triflate/caffeine‐derived salt as a catalytic system (Scheme 43). The reaction was carried out in dimethyl carbonate, for 3–48 h, at 2°C or at room temperature. The products **138** were formed in both *endo* and *exo* conformations and in yields from 20% to 99%. Additionally, the catalyst was recovered and reused up to five times, maintaining a good performance.

**SCHEME 44:**
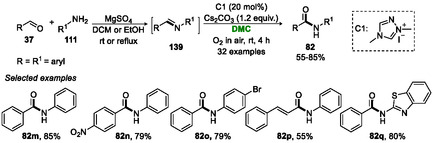
Synthesis of amides **82** in DMC as solvent [62].

In 2022, Ramarao et al. [62] published a work on an NHC‐catalyzed conversion of imines **139** into amides **82** in the presence of molecular oxygen as oxidant and dimethyl carbonate as solvent. Starting from equimolar amounts (5 mmol) of aldehydes **37** and anilines **111**, the aldimine intermediates **139** were formed either in DCM or EtOH (in the dark) at room temperature overnight or in EtOH at reflux temperature (Scheme 44). Then, without isolation, these intermediates suffered NHC‐catalyzed, with 1,4‐dimethyl‐1,2,4‐triazolium iodide (C1), imine umpolung–oxidation in the presence of 1.2 equiv. of Cs_2_CO_3_. Thus, thirty‐two amides **82** were afforded in 55–85% yield. Additionally, gram‐scale reactions (10 mmol) were conducted, and the products **82m‐o** were obtained in 70%, 65%, and 66% yield, respectively.

**SCHEME 45:**
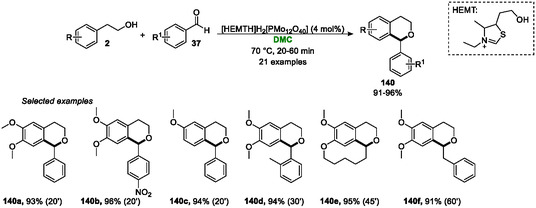
Keggin‐type heteropolyacid (HPA)‐based catalyst in the synthesis of substituted isochromanes **140** [63].

In 2021, Yang et al. [63] published a study on the preparation of various substituted isochromanes **140** from arylethanols **2** and aldehydes **37** in dimethyl carbonate as solvent and the heterogeneous green catalyst Keggin‐type heteropolyacid (HPA). The authors synthesized the catalyst [HEMTH]H_2_[PMo_12_O_40_] and used it (40 mol%, 20–60 min at 70°C) in the Oxa‐Pictet–Spengler cyclization between arylethanols **2** (0.6 mmol) and a small excess of aldehydes **37** (1.1 equiv.) (Scheme 45). The reaction was suitable for a wide range of substrates, and the products were formed in excellent yields, except in the case of phenethyl alcohol, with which the reaction did not occur. Moreover, 3 gram‐scale reactions were investigated (6 mmol), and the corresponding products **140a**‐**c** were obtained in 91%, 90%, and 93%, respectively. Additionally, the recovery and reusability of the catalyst were studied, and it was demonstrated to be efficient for eight consecutive reactions.

## 5 OCs in Other Reactions

### 5.1 Esterification Reactions

**SCHEME 46:**
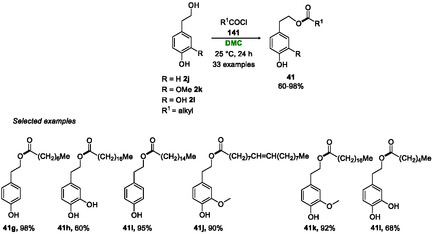
DMC as a solvent in the preparation of lipophilic products **41** [64].

In 2019, Bernini et al. [64] proposed the esterification of tyrosol **2j**, homovanillyl alcohol **2k**, and hydroxytyrosol **2l** to achieve the corresponding lipophilic products **41** in good yields (Scheme 46). Thirty‐three examples were obtained after 24 h of reaction at room temperature in the presence of an excess of the acyl chloride **141** (1.2 equiv.) and DMC as solvent. Notably, the desired products were evaluated for biological activities, and tyrosol caprylate, capriate, and laurate revealed antimicrobial effects against *Leishmania major*, *L. infantum*, *Staphylococcus aureus*, *S. xylosus*, *Bacillus cereus*, and *Brevibacterium flavum*. Hydroxytyrosol acetate showed antioxidant activity, whilehydroxytyrosol oleate exhibited anti‐inflammatory activity, and both were active against the proliferation of human cervical cells. Moreover, the plant *Olea europaea* was tested, and its hydroxytyrosol‐enriched extracts were esterified and assayed. The resulting products displayed antiproliferative activity against human colon cancer cells.

**SCHEME 47:**
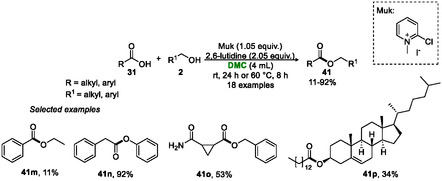
Steglich esterification of carboxylic acids **31** in DMC as solvent [65].

In 2021, Jordan et al. [65] described a Steglich esterification of carboxylic acids **31** through a green protocol, avoiding hazardous reagents, such as carbodiimide, dicyclohexylcarbodiimide, and 4‐dimethylaminopyridine, as well as solvents such as dichloromethane and *N*,*N*‐dimethylformamide. In a high‐throughput screening, Mukaiyama's reagent (Muk) and dimethyl carbonate were found to be the most effective reagent/solvent system. Thus, from equimolar amounts (2 mmol) of carboxylic acids **31** and alcohols **2** at room temperature for 24 h, or at 60°C for 8 h, in the presence of Muk (1.05 equiv.) and the base 2,6‐lutidine (2.05 equiv.), which substituted the common triethylamine, eighteen esters **41** were afforded in 11–92% yield. The reaction worked well with various substrates **31** and **2**, except when 1*H*‐pyrazole‐3‐carboxylic acid and benzyl alcohol were used as starting materials (Scheme 47).

### 5.2 Redox Reactions

**SCHEME 48:**
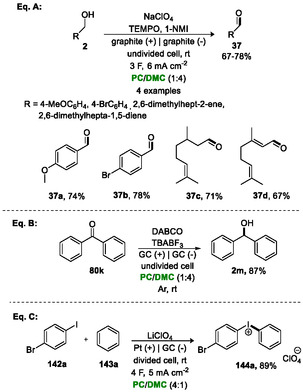
PC/DMC mixture solvent in the electrochemical synthesis of aldehydes **37** (Eq. A), alcohol **2m** (Eq. B), and diaryl iodonium salt **144a** (Eq. C) [66].

In 2025, Prudlik et al. [66] described an anodic electroorganic synthesis of diaryliodonium salts, cathodic reduction of ketones, and oxidation of alcohols using OCs as solvents. The authors justified the use of the PC/DMC binary OC system based on its high performance in energy‐storage electrolytes and the sustainability attributed to these solvents. The authors underlined that both solvents remained stable during anodic oxidation and cathodic reduction and were not affected by the applied potential. Furthermore, OCs exhibited high electrochemical stability, solubility, and dissociation from supporting electrolytes. For the oxidation of alcohols, the reaction was carried out using 0.1 mol/L of alcohols **2** as substrates, an undivided cell with graphite electrodes, three charge equivalents, a current density of 6 mA cm^−2^, 0.04 mol/L of NaClO_4_ as supporting electrolyte, and 4.5 equiv. of 1‐methylimidazole as base, 0.1 equiv. of TEMPO, and a mixture of PC/DMC (1:4) as solvent (Scheme 48, Eq. A). Under this condition, the targeted products were obtained in 67–78% yield. For the reduction of benzophenone **80k** (1 mmol), it was used an undivided cell, a glassy carbon (GC) cathode, and 3 equiv. of DABCO as a sacrificial agent (to be oxidized at the counter electrode), and 0.12 mol/L of TBABF_4_ in a 1:4 mixture of PC/DMC as solvent under an air atmosphere, affording diphenylmethanol **2m** in 87% yield (Scheme 48, Eq. B). For the synthesis of diaryl iodonium salt **144a**, the authors employed 4‐bromo‐iodobenzene **142a** and benzene **143a** as substrates, affording product **144a** in 89% of yield. The reaction was carried out applying a divided cell with platinum anode, PC/DMC ratio of 4:1 as solvent, 1 mol/L of LiClO_4_ as the supporting electrolyte, a current density of 5 mA cm^−2^, and 4F per mole of substrate (Scheme 48, Eq. C).

**SCHEME 49:**
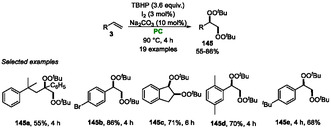
Preparation of bisperoxides **145** in PC as a solvent [67].

In 2021, Gao et al. [67] reported a protocol to access bisperoxides **145** in the presence of TBHP (3.6 mmol) as oxidant, I_2_ (0.03 mmol) as a catalyst, and Na_2_CO_3_ (0.1 mmol) as additive in propylene carbonate as solvent at 90°C for 4–6 h (Scheme 49). Various aryl alkenes **3**, bearing electron‐withdrawing and electron‐donating groups, were investigated, and all reacted smoothly. However, no reaction was observed when cyclohexene, oct‐1‐ene, allylbenzene, 2‐vinylpyridine, and 5‐methylethylene were used as substrates. The authors commented that for the first three alkenes, the cause may be the loss of resonance. Additionally, a gram‐scale reaction (from 1 to 20 mmol) was carried out, and the product **145a** was obtained in 78% yield.

**SCHEME 50:**
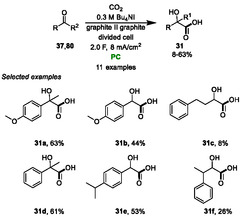
Electro‐synthesis of α‐hydroxy acids **31** [68].

In 2022, Seidler et al. [68] developed an electro‐carboxylation(graphite cathode, 8 mA/cm^2^, 2*F*, 0.3 mol/L Bu_4_NI/PC) of a variety of aldehydes **37** and ketones **80** through a direct insertion of CO_2_ (Scheme 50). The authors studied different solvents, including MeCN, DMF, DMSO, and PC, and this last furnished the best results, possibly due to its high polarity and redox stability. The products **31** were achieved in 8–63% yield.

**SCHEME 51:**
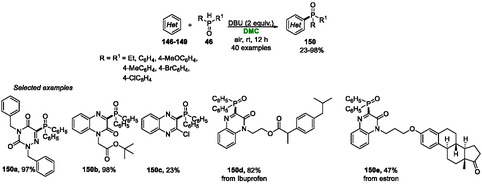
Synthesis of monophosphonated*N*‐heterocycles **150** and their applications [69].

In 2023, Cai et al. [69] described a protocol to access various monophosphonated*N*‐heterocycles **150** from different 1,2,4‐triazine‐3,5(2*H*,4*H*)‐diones **146**, quinoxalin‐2(1*H*)‐ones **147**, quinoxalines **148**, and pyrazinones **149** with phosphine oxides **46**. A total of forty products **150** were formed in 23–98% yield after 12 h of reaction at room temperature, under an air atmosphere. The optimal conditions involved the use of 0.2 mmol of substrates **146**‐**149**, 2.5 equiv. of phosphine oxides **46**, 2 equiv. of 1,5‐diazabicyclo[5,4,0]undec‐5‐ene (DBU) without metals and photocatalyst (Scheme 51). Noteworthy, this methodology did not require a further purification step; only extraction was needed, which is desirable for industrial applications. Moreover, bioactive molecules, such as vanillylacetone, vanillin, aspirin, Ibuprofen, and estrone, were subjected to this protocol and their corresponding products were generated in 47–82% yield. Unfortunately, a series of substrates weren’t responsive, such as 1‐ethylpyrido[2,3‐*b*]pyrazin‐2(1*H*)‐one, benzo[*d*]thiazole, 3‐methylquinazolin‐4(3*H*)‐one, 2*H*‐benzo[*b*][1,4]oxazin‐2‐one, 1‐methylpyrazin‐2(1*H*)‐one, 1‐methyl‐1*H*‐benzo[*d*]imidazole, 1‐methyl‐1*H*‐indole, 1*H*‐purine, quinoline, isoquinoline, phthalazine, and quinazoline. Furthermore, a gram‐scale reaction was carried out, affording product **150a** in 92% yield.

**SCHEME 52:**
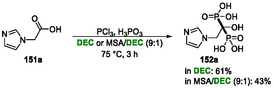
Synthesis ofzoledronic acid **152a** [70].

In 2021, Grün et al. [70] described the application of diethyl carbonate and its mixture with methanesulfonic acid (MSA) as a solvent in the preparation of zoledronic acid **152a** from imidazole‐1‐yl‐acetic acid **151a** and phosphorus trichloride/phosphorous acid. The authors commented that even if the protocol was less efficient than the one reported in the literature with sulfolane as solvent, the new solvents investigated were more environmentally friendly than the commonly used ones, such as MSA and sulfolane. The best reaction conditions were found to be 3.2 equiv. of phosphorus trichloride and 3.1 equiv. of phosphorous acid at 75°C for 3 h. The product **152a** was obtained in 61% yield when used only DEC as solvent, and in 43% yield using a mixture of MSA:DEC = 9:1 (Scheme 52).

### 5.3 Multicomponent Reactions

**SCHEME 53:**
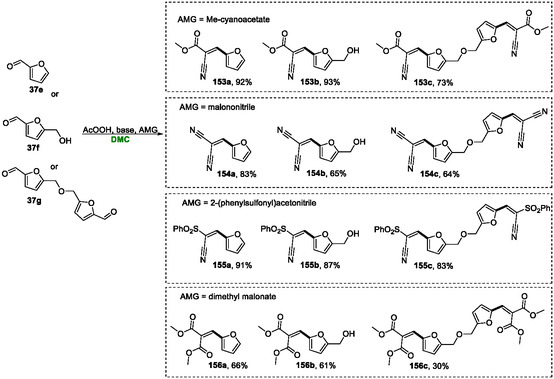
DMC as a solvent in the Knoevenagel condensation reactions [71].

Annatelli et al. [71] described for the first time the Knoevenagel condensation of furfural **37e**, 5‐hydroxymethyl‐2‐furfural **37f** (HMF), and 5,5′‐oxy(bismethylene)‐2‐furaldehyde **37g** (OBMF) in dimethyl carbonate as green solvent in the presence of four types of active methylene groups (AMG): Me‐cyanoacetate, malononitrile, 2‐(phenylsulfonyl)acetonitrile, and dimethyl malonate (Scheme 53). From their study, with Me‐cyanoacetate, for **37e** and **37f**, the base 1,4‐diazabicyclo[2.2.2]octane (DABCO) worked smoothly; for furfural **37e**, the same results were achieved also using pyrrolidine (Py) and *N*‐methylpyrrolidine (NMPy). The reactions were conducted at room temperature for 30 min (for **37e** and **37f**) and 1 h (for **37g**), and the corresponding products **153a**‐**c** were reached in 92%, 93%, and 73% yield, respectively. When the AMG malononitrile was used, for all substrates **37** the base was DABCO, at room temperature, and the corresponding products **154a**‐**c** were formed in 83%, 65%, and 64% yield, respectively. With 2‐(phenylsulfonyl)acetonitrile, at the same reaction conditions, the product **155a** was produced in 91%, **155b** in 87%, and **155c** in 83% yield. Finally, using dimethyl malonate as AMG, the used base was pyrrolidine, and the compound **156a** was furnished at 100°C after 3 h in 66% yield in dimethyl carbonate, as well as in 2‐MeTHF and cyclopentyl methyl ether as solvents. Under the same reaction conditions, products **156b** and **156c** were obtained in 61% and 30% yield (this last was calculated by ^1^H‐NMR), respectively.

**SCHEME 54:**
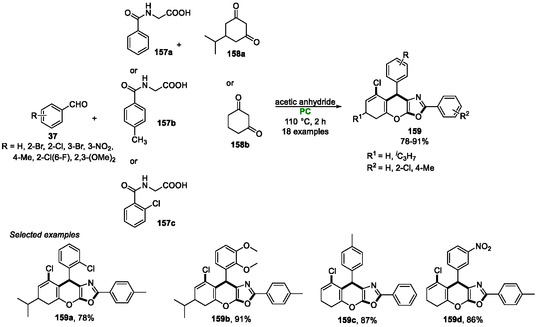
Synthesis of new chromeno[3,2‐*d*]oxazoles **159** [72].

In 2019, Zamani et al. [72] published a one‐pot three‐step synthesis of new chromeno[3,2‐*d*]oxazoles **159** in propylene carbonate. Eighteen examples of this class of compound were achieved in 78–91% yield, after 2 h of reaction at 110°C. The optimal conditions involve stirring equimolar amounts of different arylaldehydes **37**, hippuric acids **157**, acetic anhydride, and 1,3‐cyclohexanediones **158** in the presence of calcium acetate (0.01 equiv.) (Scheme 54).

### 5.4 Cascade Reactions

**SCHEME 55:**
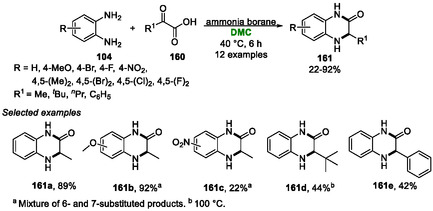
Synthesis of 3,4‐dihydroquinoxalin‐2(1*H*)‐ones **161** through a catalyst‐free cascade amination/cyclization/reduction reaction [73].

In 2024, Zhao et al. [73] reported a catalyst‐free cascade amination/cyclization/reduction reaction for the synthesis of 3,4‐dihydroquinoxalin‐2(1*H*)‐ones **161** from 1,2‐diaminobenzenes **104** and keto‐acids **160**, with ammonia borane (AB, NH_3_BH_3_) as a hydrogen‐donor and DMC as an eco‐friendly solvent. The best reaction conditions were determined using 0.5 mmol of **104**, 1.5 equiv. of **160**, and 2 equiv. of AB in DMC at 40°C for 6 h. The authors have prepared twelve different examples in 22–92% yield, which were determined by NMR spectroscopy using DMF as an internal standard. To demonstrate the applicability of this methodology, the authors conducted a gram‐scale synthesis (10 mmol) of the target 3,4‐dihydroquinoxalin‐2(1*H*)‐one **161a**, which was affordedin 65% yield after 24 h of reaction (Scheme 55).

**SCHEME 56:**
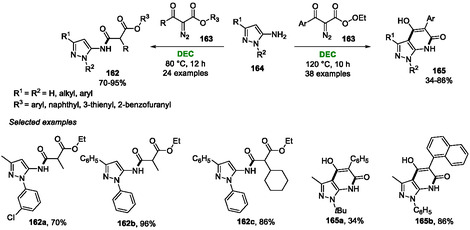
Preparation of 4‐hydroxy‐pyrazolo[3,4‐*b*]pyridin‐6‐ones **165** and *N*‐pyrazole amides **162** through a cascade Wolff rearrangement/acylation [74].

In 2021, Zhang et al. [74] reported a cascade Wolff rearrangement/acylation to access 4‐hydroxy‐pyrazolo[3,4‐*b*]pyridin‐6‐ones **165** and *N*‐pyrazole amides **162** from 5‐aminopyrazoles **164** and α‐diazo ketones **163**. When α‐diazo ketones bearing an ethoxy substituent were used, the best reaction conditions involved reacting **162** with 1.2 equiv. of reagent **163**, at 120°C for 10 h. In this case, the authors highlighted that the products did not require purification steps, but only recrystallization and filtration. Instead, with the other α‐diazo ketones, the reaction was conducted at 80°C for 12 h (Scheme 56). With this protocol, it was possible to generate sixty‐two examples of compounds **162** and **165** in 34–95% yield. The reaction did not work with substrates ethyl 2‐diazo‐3‐oxo‐3‐(*o*‐tolyl)propanoate and 1‐(*tert*‐butyl)‐3‐methyl‐1*H*‐pyrazol‐5‐amine.

### 5.5 Miscellaneous

**SCHEME 57:**
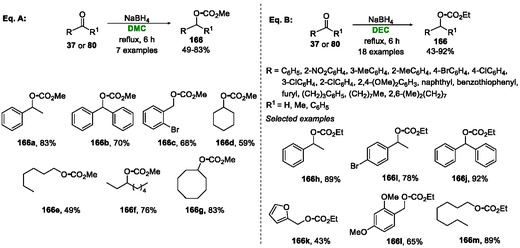
DMC and DEC as solvents in the preparation of carbonates **166** [75].

In 2019, Osumah et al. [75] reported an alternative to the traditional two‐step sequence to prepare carbonates **166**. The authors described a one‐step sequence from ketones **80** and aldehydes **37** via treatment with NaBH_4_ in dimethyl (DMC) and diethyl carbonate (DEC) solvents at reflux temperatures (Scheme 57). The reaction was promoted using 1 mmol of carbonyl compound **80** or **37** in the presence of 1 equiv. of NaBH_4_, DMC as solvent at reflux temperature during 6 h, giving the desired carbonates **166**, which had the ‐CO_2_Me moiety in their structure (Scheme 57, Eq. A). Also, the protocol was extended to use DEC as a solvent, providing the respective carbonates with the ‐CO_2_Et moiety in their structure. Through this methodology, it was possible to obtain seven examples in 49–83% yield using DMC and eighteen carbonates **166** in 43–92% yield using DEC (Scheme 57, Eq. B).

**SCHEME 58:**
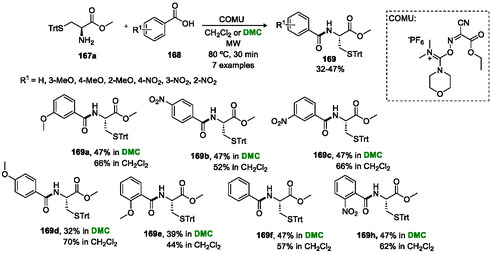
Synthesis of novel peptidomimetics arylamides **169** in DMC as solvent [76].

In 2020, Serralbo et al. [76] published an alternative methodology employing DMC in the synthesis of novel peptidomimetics, arylamides **169**. In this study, the optimal reaction condition was carried out with 0.08 mmol of *S*‐trityl‐L‐cysteine **167a**, 0.08 mmol of benzoic acids **168**, 0.08 mmol of [(1‐cyano‐2‐ethoxy‐2‐oxoethylidenaminooxy) dimethylamino‐morpholino carbenium hexafluorophosphate] (COMU) as coupling reagent, in 5 mL of CH_2_Cl_2_ or DMC, in a sealed tube under microwave irradiation at 80°C for 30 min.The desired products were obtained in moderate to excellent yields (Scheme 58). Although dimethyl carbonate afforded lower yields, the authors emphasized that isolating the products was difficult when using CH_2_Cl_2_, since the byproducts are quite soluble in this solvent. Notably, dimethyl carbonate proved to be a good green solvent alternative.

**SCHEME 59:**
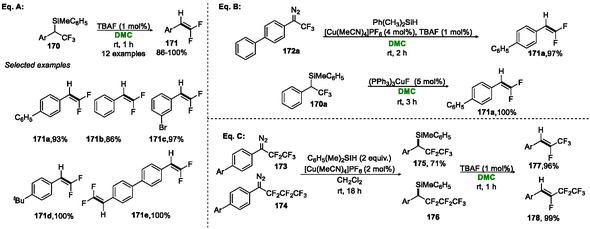
Synthesis of 1‐aryl‐2,2‐difluoroalkenes **171** (Eqs. A and B), perfluoroalkyl organosilanes **175**, **176**, and (*Z*)‐1‐benzylideneperfluoroalkenes **177**, **178** (Eq. C) [77].

Carreras and Ollevier [77] proposed the one‐pot synthesis of 1‐aryl‐2,2‐difluoroalkenes **171** starting from silanes **170** (Scheme 59, Eq. A). The reaction solvent was dimethyl carbonate, which was demonstrated to be a valid alternative to dichloromethane, the comparative solvent used in this work. After 1 h of reaction at room temperature in the presence of 1 mol% of tetra‐*n*‐butylammonium fluoride (TBAF), twelve examples were prepared in good to quantitative yields, which were determined by ^19^F NMR. Moreover, the reaction between 4‐(1‐diazo‐2,2,2‐trifluoroethyl)−1,1′‐biphenyl **172a** and Ph(CH_3_)_2_SiH in the presence of 1 mol% of TBAF and 2 mol% of [Cu(CH_3_CN)_4_]PF_6_ in dimethyl carbonate at room temperature for 2 h afforded 4‐(2,2‐difluorovinyl)−1,1′‐biphenyl **171a**, in 97% yield (Scheme 59, Eq. B). When the substrate **170a** was reacted with 5 mol% of (PPh_3_)_3_CuF, the product **171a** was achieved quantitatively. Additionally, the authors demonstrated for the first time the reaction of diazo compounds **173**, **174** to give the perfluoroalkyl organosilanes **175**, **176,** and then, the (*Z*)‐1‐benzylideneperfluoroalkanes **177**, **178** (Scheme 59, Eq. C).

**SCHEME 60:**
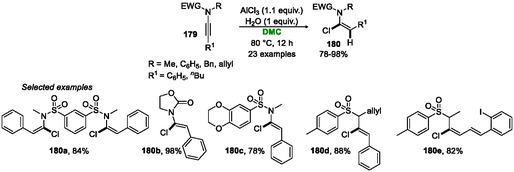
DMC as solvent in the preparation of (*E*)‐α‐chloroenamides **180** [78].

In 2021, Alla et al. [78] developed a strategy to access (*E*)‐α‐chloroenamides **180** via chlorination with AlCl_3_ in dimethyl carbonate, avoiding chlorinated solvents. A total of twenty‐six enamides **180** were obtained in 78–98% yield through the reaction of ynamides **179** with AlCl_3_ (1.1 equiv.) in the presence of an equimolar amount of water at 80°C for 12 h, (Scheme 60). Also, a bis‐hydrochlorination of the diyne could be possible by using 2 equiv. of water and 2.2 equiv. of AlCl_3_, and the product **180a** was reached in 84%.

**SCHEME 61:**
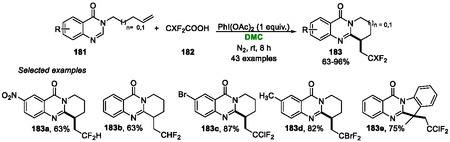
Synthesis of CHF_2_/CClF_2_/CBrF_2_‐substituted ring‐fused quinazolinones **183** [79].

In 2022, Gui et al. [79] published a visible light‐promoted protocol to afford CHF_2_/CClF_2_/CBrF_2_‐substituted ring‐fused quinazolinones **183** from alkenyl‐substituted quinazolinones **181**. A wide range of substrates was applied (0.2 mmol), 5‐, 7‐, and 8‐substituted quinazolin‐4(3*H*)‐one, including functional groups at the position 6, electron‐neutral, ‐donating, or weakly electron‐deficient, and strong electron‐withdrawing groups at the 6‐position, besides disubstituted ones. All substrates reacted smoothly with difluoroacetic acid **182** in the presence of PhI(OAc)_2_ (1 equiv.) in dimethyl carbonate, under nitrogen atmosphere, at room temperature for 8 h (Scheme 61). The authors commented that only traces were observed when 2‐difluoro‐2‐phenyl acetic acid was employed.

**SCHEME 62:**
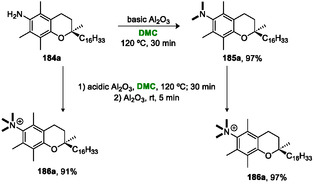
DMC as solvent and methylating agent in the synthesis of *N*‐methylated derivatives of α‐tocopheramine [80].

In 2022, Patel and Rosenau [80] described the synthesis of *N‐*methylated derivatives of α‐tocopheramineusing dimethyl carbonate as both solvent and methylating agent. The reaction was carried out by applying 1 mmol of α‐tocopheramine **184a**, 50 mL of dimethyl carbonate, 5 equiv. of basic Al_2_O_3_ as a catalyst, under an inert atmosphere and conventional heating for 30 min, achieving the product **185a** in 97% yield (Scheme 62). Under the optimal condition, with the use of acidic aluminum oxide instead of basic one and adding another five mass equivalents of Al_2_O_3_ after cooling the reaction mixture, the starting amine **184a** was quaternized to *N*,*N*,*N*‐trimethyl‐α‐tocopherol ammonium **186a** in 91% yield. Alternatively, the same product can be obtained in 97% yield starting from compound **186a**.

**SCHEME 63:**
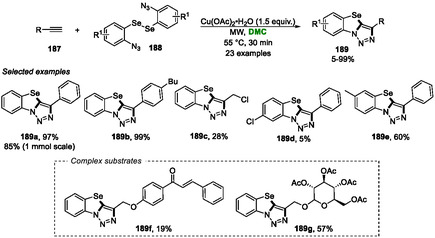
Preparation of fused benzo[4,5][1,3]selenazolo[3,2‐*c*][1,2,3]triazoles **189** through a CuAAC intramolecular selenylation [81].

Teixeira et al. [81] proposed a copper‐mediated domino CuAAC intramolecular selenylation for the synthesis of fused benzo[4,5][1,3]selenazolo[3,2‐*c*][1,2,3]triazoles **189** from terminal alkynes **187** and 1,2‐bis(2‐azidoaryl)diselenides **188** under microwave irradiation using dimethyl carbonate as solvent (Scheme 63). The best reaction conditions were found to be a mixture of alkyne (0.15 mmol) **187**, diselenide (1 equiv.) **188**, and Cu(OAc)_2_·H_2_O (1.5 equiv.) in DMC under microwave irradiation at 55°C for 30 min. To demonstrate the generality of this methodology, the authors tested the reaction using different terminal alkynes and diselenides, and twenty‐three products were obtained in 5–99% yield. Moreover, to prove the functional compatibility of the method, complex terminal alkynes derivatives from important bioactive compounds were successfully used as starting materials, affording five examples in 19–88% yield. Additionally, a 1 mmol scale reaction was carried out, and product **189a** was furnished in 85% yield.

**SCHEME 64:**
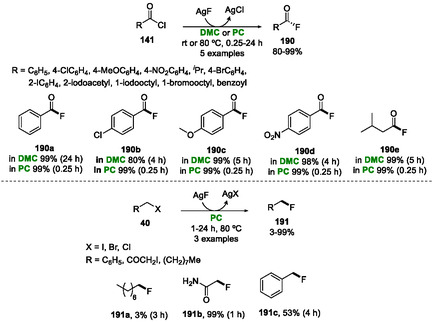
DMC and PC as solvents in the fluorination of aromatic and aliphatic acyl chlorides [82].

In 2023, Abellán et al. [82] described the fluorination of aromatic and aliphatic acyl chlorides **141** by employing dimethyl carbonate and propylene carbonate as bio‐based solvents. The authors optimized the reaction conditions applying 5 mol% of 1‐((diphenylphosphaneyl)methyl)‐1*H*‐benzo‐1,2,3‐triazole as catalyst, 2.5 equiv. of AgF in dichloromethane at room temperature for 2.5 h (Scheme 64). After a previously determined condition, the authors carried out a study of different OCs as solvents, which are considered more sustainable options. Among the evaluated solvents, DMC and PC presented excellent yields. By employing them, it was possible to increase the temperature to 80°C, longer reaction times were needed compared to CH_2_Cl_2_. After that, the procedure was evaluated with a variety of acyl chlorides, giving the desired products **190** in good to excellent yields. Furthermore, when the authors varied the alkyl halides, CH_2_Cl_2_, DMF, and PC were used as solvents. The products **191a** and **191b** could be obtained only when PC was used as solvent, being isolated in 3% and 99% yield, respectively.

**SCHEME 65:**
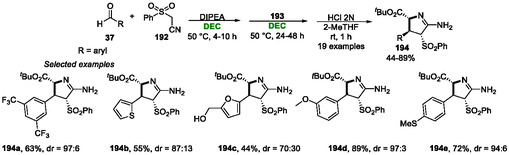
Preparation of cyclic amidines **194** in DEC as a solvent [83].

In 2021, Meninno et al. [83] proposed a new methodology to synthesize cyclic amidines **194** with three stereogeniccenters in good to high yields and with high diastereoselectivity. In this one‐pot process, two green and biodegradable solvents, diethyl carbonate and 2‐methyl tetrahydrofuran, were employed without metals, through a three‐step reaction (Scheme 65). First, a Knoevenagel condensation occurred between 0.15 mmol of both substrates, **37** and **192**, with a base *N*,*N*‐diisopropyl ethylamine (DIPEA) in diethyl carbonate at 50°C for 4–10 h, to form the intermediate alkyne species. After the disappearance of the starting materials, 0.18 mmol of the Schiff base *tert*‐butyl ester glycinate benzophenone **193** was added and reacted with the alkyne through a Michael addition, in diethyl carbonate at 50°C for 24–48 h. Once the reaction is completed, the solvent was removed and 2‐methyl tetrahydrofuran and HCl 2 mol/L were added to give the last step of hydrolysis and cyclization. Nineteen cyclic amidines **194**, bearing electron‐withdrawing and electron‐donating groups, cyclic and heterocyclic substituents, with the majority of *trans*,*trans* diastereoisomer, were reached in 44–89% yield. Additionally, a gram‐scale reaction was studied, using 1g of phenyl sulfonylacetonitrile reagent and affording the corresponding productin 60% yield, only *trans*,*trans* diastereoisomer.

**SCHEME 66:**
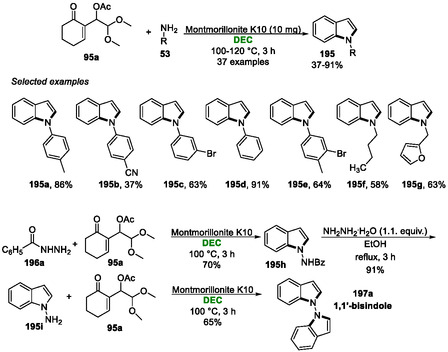
Montmorillonite K10‐catalyzed synthesis of *N*‐substituted indoles **195** [84].

In 2023, Han et al. [84] employed diethyl carbonate as green solvent in the reaction between a wide range of primary amines **53**, aromatic and aliphatic ones, and MBH acetate of cyclohexenone **95a**. Montmorillonite K10 was used as a catalyst at 100–120°C during 3 h, affording thirty‐seven *N*‐substituted indoles **195** in 37–91% yield (Scheme 66). Moreover, the catalyst was recovered and reused three times without decreasing the yield. Besides, a gram‐scale reaction of 10 mmol was performed and afforded the product **195a** in 80% yield. Furthermore, the authors applied their methodology to prepare a bacterial biofilm inhibitor, 1,1′‐bisindole **197a**, in 65% yield, through the reaction between **196a** and MBH acetate of cyclohexenone **95a**.

## 6 Conclusions and Outlooks

The purpose of this review was to demonstrate the relevance of dimethyl carbonate (DMC), diethyl carbonate (DEC), and propylene carbonate (PC) as solvents that have indeed shown the ability to replace petroleum‐based solvents, moving beyond being merely a promising alternative. The potential of OCs to establish themselves as common solvents available on the shelves of research laboratories and industrial plants was demonstrated by their efficiency in acting in various transformations, including *N*‐alkylation reactions, photoinduced transformations, hydroformylation of terpenoids, and organic electrosynthesis. Although they are not inert under all reaction conditions, which is an issue with most slightly polar solvents, OCs are considered as effective alternatives to traditional solvents. This makes various methodologies viable and promotes high performance, selectivity, and scalability. In addition to their low environmental impact, OCs, with their high biodegradability and low toxicity, also add value to carbon dioxide as a chemical input and are considered negative‐carbon chemicals. Therefore, the benefits of OCs exceeded simply replacing hazardous substances; they stand out for their high biodegradability rate, low toxicity, and economic viability, allowing for safer processes and thus contributing to environmental sustainability. In this context, the growing use of sustainable alternatives to conventional solvents indicates not only a current trend in industrial development but also an increasing need for sustainable chemistry.

## Author Contributions

Gabriela T. Quadros and Livia C. L. Valente wrote the original draft, edited, and visualized the review. Thiago Barcellos and Daniela Hartwig wrote, visualized, and edited the review. Laura Abenante and Eder J. Lenardão conceived, wrote, edited, and visualized the review.

## Funding

This work was supported by the Coordenação de Aperfeiçoamento de Pessoal de Nível Superior (001), Conselho Nacional de Desenvolvimento Científico e Tecnológico, Fundação de Amparo à Pesquisa do Estado do Rio Grande do Sul, and Financiadora de Estudos e Projetos (CT‐INFRA).

## Conflicts of Interest

The authors declare no conflicts of interest.
